# Identification of Two Evolutionarily Conserved 5' *cis*-Elements Involved in Regulating Spatiotemporal Expression of *Nolz-1* during Mouse Embryogenesis

**DOI:** 10.1371/journal.pone.0054485

**Published:** 2013-01-22

**Authors:** Sunny Li-Yun Chang, Ya-Chi Liu, Shih-Yun Chen, Ting-Hao Huang, Pei-Tsen Liu, Fu-Chin Liu

**Affiliations:** 1 Institute of Molecular Systems Biomedicine, China Medical University, Taichung, Taiwan, Republic of China; 2 Institute of Neuroscience, National Yang-Ming University, Taipei, Taiwan, Republic of China; The Walter and Eliza Hall of Medical Research, Australia

## Abstract

Proper development of vertebrate embryos depends not only on the crucial funtions of key evolutionarily conserved transcriptional regulators, but also on the precisely spatiotemporal expression of these transcriptional regulators. The mouse *Nolz-1*/*Znf503/Zfp503* gene is a mammalian member of the conserved zinc-finger containing NET family. The expression pattern of *Nolz-1* in mouse embryos is highly correlated with that of its homologues in different species. To study the spatiotemporal regulation of *Nolz-1,* we first identified two evolutionarily conserved *cis*-elements, UREA and UREB, in 5′ upstream regions of mouse *Nolz-1* locus. We then generated UREA-*LacZ* and UREB-*LacZ* transgenic reporter mice to characterize the putative enhancer activity of UREA and UREB. The results indicated that both UREA and UREB contained tissue-specific enhancer activity for directing *LacZ* expression in selective tissue organs during mouse embryogensis. UREA directed *LacZ* expression preferentially in selective regions of developing central nervous system, including the forebrain, hindbrain and spinal cord, whereas UREB directed *LacZ* expression mainly in other developing tissue organs such as the *Nolz-1* expressing branchial arches and its derivatives, the apical ectodermal ridge of limb buds and the urogenital tissues. Both UREA and UREB directed strong *LacZ* expression in the lateral plate mesoderm where endogenous *Nolz-1* was also expressed. Despite that the *LacZ* expression pattern did not full recapitulated the endogenous *Nolz-1* expression and some mismatched expression patterns were observed, co-expression of *LacZ* and *Nolz-1* did occur in many cells of selective tissue organs, such as in the ventrolateral cortex and ventral spinal cord of UREA-LacZ embryos, and the urogenital tubes of UREB-LacZ embryos. Taken together, our study suggests that UREA and UREB may function as evolutionarily conserved *cis*-regulatory elements that coordinate with other *cis*-elements to regulate spatiotemporal expression of *Nolz-1* in different tissue organs during mouse embryogenesis.

## Introduction

*Nolz-1*, the *Drosophila* nocA like zinc-finger gene-1 (also know as *Znf503* or *Zfp503* by Genome Informatics), is a member of the SP1-related *nocA/elb/tlp-1* (NET) family. Members of the NET family include *Nolz-1* and *Znf703/Zfp703* in mammals, *nlz1* and *nlz2* in *Danio rerio* (zebrafish), *nocA* and *elbow* (*elB*) in *Drosophila melanogaster* (fruit fly) and *tlp-1* gene in *Caenorhabditis elegans* (nematode worm) [1]. The zebrafish *nlz2* and *nlz1* genes are orthologues of *Nolz-1* and *Zfp703*, respectively. These two paralogues genes are identified in many vertebrates whose genome has been sequenced and annotated. Studies show that the NET family proteins function as transcriptional regulators that play crucial roles in patterning and development of many embryonic tissue organs, except Zfp703 whose role in embryonic development has not yet been uncharacterized. Notably, *ZNF703* has recently been identified as an oncogene in human breast tumors [2], [3]. *Tlp-1* as an asymmetric cell fate determinant is involved in control of tail tip morphogenesis in male *C. elegans* worm [4]. *ElB* and *nocA* define the appendage-forming domain of imaginal discs and regulate the proximal-distal axis of *Drosophila* wings [5]. *ElB* and *nocA* are also involved in regulating proper development of eyes, brain and tracheal branches (lung) in *Drosophila* [6], [7], [8]. The two zebrafish *nlz* genes are expressed in spinal cord, specific rhombomeres of hindbrain and the midbrain-hindbrain boundary (MHB) at the neural tube stages. *Nlz2* is also expressed in the forebrain region of zebrafish embryos [9]. Mis-expression and ectopic expression of *nlz* genes cause abnormal rhombomere identities that implicate important roles of *nlz* genes in early hindbrain development [9], [10], [11]. Furthermore, both *nlz1* and *nlz2* are also involved in directing proper closure of the optic fissure, and thus play crucial roles for zebrafish eye development [12].

Expression of mouse *Nolz-1* gene is detected in many developing tissue organs, including the forebrain, hindbrain and spinal cord of the embryonic central nervous system, eyes, lung and limbs, where aforementioned *Nolz-1* homologues in *Drosophila* and zebrafish are also expressed [13], [14], [15], [16]. In the developing nervous system, *Nolz-1* is highly expressed in differentiating neurons of striatum in telencephalon [13]. *Nolz-1* is also expressed in rhombomeres 3, 5 and more caudal levels of the segmented early developing hindbrain, and later on in specific longitudinal columns and clusters, including the somatic motor neurons of hypoglossal nucleus in embryonic hindbrain [14]. In the developing chick spinal cord, *Nolz-1* has been shown to regulate development of subtypes of motor neurons [16]. In developing eyes, in addition to be expressed in the mouse optic fissure tissue as *nlz* genes expression in developing zebrafish eyes, *Nolz-1* is preferentially expressed in the retina pigment epithelial cells [12], [14].

During early limb development, *Nolz-1* is preferentially expressed in apical ectodermal ridge (AER) and *zone* of polarizing activity (ZPA), two signaling centers for limb bud patterning, at early stages of limb bud formation and later in restricted domains of limb bud mesenchymal tissues [15]. This expression pattern implicates a conserved role of *Nolz-1* and its *Drosophila* homologues in formation and patterning the appendages [5], [15]. *Nolz-1* is also expressed in other embryonic tissues, such as lateral plate mesoderm, olfactory vesicles, otic vesicles, branchial arches and its derivatives [15].

Because the expression patterns and functions of *Nolz-1* and its homologues are evolutionarily conserved among different species, we postulated that the transcriptional regulation mechanisms of *Nolz-1* homologues might be conserved during evolution. By genomic sequence comparison of non-coding regions among different *Nolz-1* homologoues loci, we identified several vertebrate-conserved *cis*-elements in the mouse *Nolz-1* gene locus. To further elucidate the potential roles of the conserved elements *in vivo*, we generated several lines of *LacZ* reporter mice carrying different conserved *cis*-elements of mouse *Nolz-1*.

In the present study, we report the characterization of two conserved potential 5′ upstream regulatory elements, UREA and UREB, of mouse *Nolz-1* gene. We found that both UREA and UREB were capable of functioning as tissue-specific enhancers to direct the *LacZ* reporter gene expression in specific tissue organs of mouse embryos. The UREA element effectively directed *LacZ* reporter gene expression in the developing central nervous system, including selective regions in the forebrain, midbrain, the segmented developing hindbrain and spinal cord in which endogenous *Nolz-1* was expressed. Compared to the UREA element, the UREB element directed *LacZ* expression mainly in other tissue organs, including the *Nolz-1* expressing AER of limb buds, the branchial arches and the urogenital organs. Double labeling of X-gal and *Nolz-1* confirmed co-expression of *Nolz-1* and *LacZ* in some tissue organs, though mismatched patterns were observed, suggesting that UREA and UREB *cis*-elements are important for regulating *Nolz-1* expression in specific regions of developing mouse embryos. Nonetheless, other regulatory elements were also required for fully recapitulate endogenous *Nolz-1* expression during embryogenesis.

## Methods

### Ethics Statement

The germ-line transmitted transgenic mice were maintained in the Animal Centers of National Yang-Ming University (NYMU) and China Medical University (CMU). The animal protocols were approved by Animal Care and Use Committees of NYMU and CMU.

### Generation of UREA-LacZ and UREB-LacZ Transgenic Mice

The p1229-UREA-LacZ-2XHS4 and p1229-UREB-LacZ-2XHS4 transgenic plasmids for generating UREA-LacZ and UREB-LacZ transgenic mice, respectively, were constructed using the p1229 plasmid, which contained a human β-globin minimal promoter and the *lacZ* reporter gene (Kindly provided by Dr. Marck Ekker of University of Ottawa, Ottawa, Canada) as backbone [17]. Subclonning was performed using either a PCR-based approach or convenient restriction enzyme cloning sites. Specific primers (UREA-51∶5′-ccctccatttaccggtctcc-3′, UREA-31∶5′-agtgggtgagtgatgagggc-3′; UREB-51∶5′-ccagtccaggactcttagg-3′, UREB-31∶5′-aaaagggtgtgggcatggc-3′) were used for generating the UREA and UREB fragments which were then subcloned into the ScaI/NotI and XbaI/SpeI sites of the p1229 plasmid. A direct repeat of a 1.2-kb fragment containing the DNase I-hypersensitivesite 4 of the chicken β-globin locus (HS4) fragment was released from SalI and XbaI sites of pJC13-1-5′HS4 (kindly provided by Dr. T.-F. Tsai of National Yang-Ming University, Taipei, Taiwan), and was then subcloned downstream the LacZ expression cassette in a reverse orientation to avoid *position silence effects* on *transgene* [18]. The transgenic fragments were released by ScaI and XhoI sites for C57/BL6 pronuclei microinjection (performed in the Transgenic Mouse Core Facility, National Taiwan University, Taipei, Taiwan). The potential founder mice were screened by PCR using tail DNA as template and HS4 specific primers (HS4-51∶5′-cctccttgggcaacctgttcag-3′; HS4-31∶5′-atgtggcactgagggacatggc-3′). Germ-lines transmitted UREA-LacZ and UREB-LacZ founder lines were identified by intercrossing with wild type mice.

### Animal Breeding and Genotyping

Transgenic reporter mice were maintained as heterozygotes. Genotypes of the transgenic embryos were analyzed by PCR using aforementioned HS4-51 and HS4-31 primers. The day of positivity of vaginal plug was defined as embryonic day (E) 0.5. At least three litters were analyzed per experiment.

### X-gal Staining, Immunohistochemistry and *in situ* Hybridization

E8.5∼E13.5 transgenic embryos and dissected embryonic tissues for whole-mount X-gal staining were fixed by 2% formaldehyde/0.2% glutaraldehyde in PBS at 4°C for different time (5∼20 minutes for E7.5∼E10.5, 30∼50 minutes for E11.5∼13.5). After washed with PBS, the embryos and tissues were immersed in solution containing 1 mg/ml X-gal/100 mM NaPO4 pH7.3/13 mM MgCl_2_/3 mM K_3_Fe(CN)_6_/3 mM K_4_Fe(CN)_6_ at 37°C for X-gal staining. The well-stained embryos were post-fixed with 4% paraformaldehyde (PFA) in PBS at room temperature for several hours or overnight (for E11.5∼E13.5). Some of them were cryosectioned after cryoprotection with 30% sucrose in PBS overnight. Some of the X-gal stained sections were further processed for nucleus fast red counter staining or immunohistochemistry as previously described [13]. Affinity-purified polyclonal rabbit anti-Nolz-1 antiserum (1∶500, generated by Dr. F.-C. Liu) rabbit polyclonal anti-Isl1/2 antiserum (1∶1,000, Abcam), rabbit polyclonal anti-Hox2b antiserum (1∶500, Covance, Emeryville, CA) and mouse monoclonal anti-β-galactosidase antibody (1∶1,000, Promega, Madison, WI) were used. *In situ* hybridization was performed as previously described [13]. Riboprobes of *LacZ* (a 1.1 kb ClaI∼SacI fragment at coding region) and *Nolz-1* (NM145459, 2640–3744) were used. For double immunohistochemistry and *in situ* hybridization, immunohistochemical staining was performed after *in situ* hybridization.

## Results

### Identification of Two Evolutionarily Conserved Up-stream Cis-elements, UREA and UREB, in the Mouse *Nolz-1* Gene Locus

**Figure 1:**
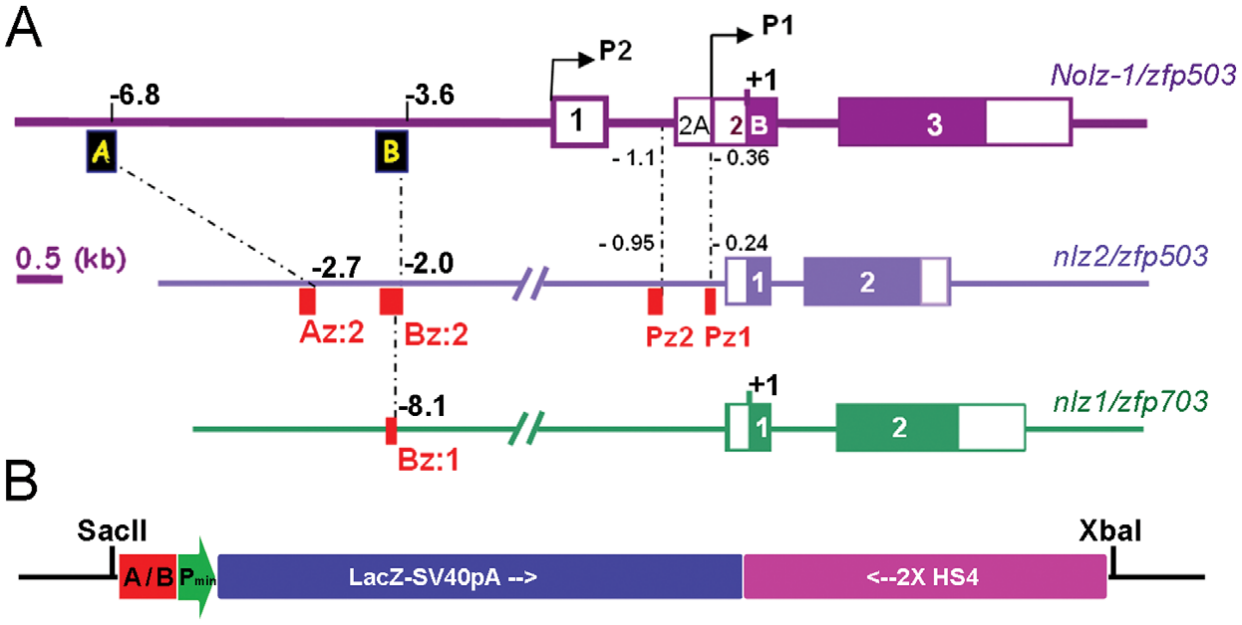
Evolutionarily conserved up-stream *cis*-elements of mouse *Nolz-1/Zfp503* gene and the UREA-LacZ and UREB-LacZ transgenic constructs. A: Diagrams show the locations of the UREA (A) and UREB (B) (black boxes) conserved upstream *cis*-elements of mouse *Nolz-1/zfp503* and their relative conserved regions in zebrafish *nlz2/zfp503* (red boxes, Az:2, Bz:2) and *nlz1/zfp703* loci (red box, Bz:1). Pz1 and Pz2 (red boxes) are another two zebrafish/mouse conserved *cis*-elements identified in the potential promoter region of *nlz2/zfp503*. Numbers indicate the distances (in kb) relative to the translation start condon (+1). B: The UREA-LacZ and UREB-LacZ transgenic constructs. The UREA or UREB (A/B) elements were inserted upstream to a minimal promoter (Pmin) following by *LacZ* expression cassette (LacZ-SV40pA) and two direct repeats of insulators (2XHS4). The SacII and XbaI sites were used to release the transgenic fragments from the vector.

The mouse *Nolz-1* gene was consisted of three exons. The coding sequences of *Nolz-1* resided in second and third exons (Figure 1A). The finding of two alternative *Nolz-1* transcripts, which were encoded by exons 1, 2A/B and 3 (NM_145459.3) or exons 2B and 3 (AY281290.1) in NCBI databank implicates two distinct promoters in the *Nolz-1* gene Locus. The BLAST analysis of the potential promoter regions against the NCBI genomic databanks, identified two small *cis*-elements which were highly conserved between *Danio rerio* (zebrafish) and mouse (Figure 1A, Pz1 and Pz2). The BLAST analysis of twenty kilobase pairs (kb) more upstream sequences further identified another two evolutionarily conserved *cis*-elements, which we named upstream-regulatory element A and B (abbreviated as UREA and UREB, respectively) in positions at −6.8 kb and −3.6 kb upstream relative to the translational start ATG codon of *Nolz-1* (Figure 1A).

The UREA and UREB 5′ *cis*-elements of mouse *Nolz-1* shared high identities (87∼97% and 86∼98%, respectively) with its orthologues in different species, including *Homo sapiens* (human), *Pan troglodytes* (chimpanzee), *Rhesus macaque* (monkey), *Bos taurus* (cow), Canis familiaris (dog), *Rattus norvegicus* (rat) and *Gallus gallus* (chicken) (data not shown). Within the 355 base pairs (bp) of UREA element, the 185^th^ to 354^th^ nucleotides (nt) were 87% identical to upstream genomic sequences of the *Nolz-1* zebrafish orthologue, *nlz2* gene (Figure 1A and Figure S1). Within the 376 bp of UREB element, the 53^th^ to 305^th^ nucleotides were 87% identical to the upstream genomic sequences of zebrafish *nlz2* which represented a zebrafish/mouse conserved UREB domain (Figure 1A and Figure S2). Interestingly, within this domain, the 81^th^ to 189^th^ nucleotides were also found conserved in the zebrafish *nlz1* gene locus (Figure S2).

### Generation of UREA-LacZ and UREB-LacZ Transgenic Reporter Mice

To elucidate the potential roles of the evolutionarily conserved UREA and UREB *cis*-elements of *Nolz-1 in vivo*, we generated UREA-LacZ and UREB-LacZ transgenic mice. The UREA and UREB DNA fragments were inserted upstream the human β-globin minimal promoter and β-galactosidase encoded *LacZ* gene following by two direct repeats of insulator to avoid potential position silencing effects (Figure 1B).

Three lines of UREA-LacZ founder mice, #21, #22 and #26, were germ-line transmitted to first generation (F1). Unfortunately, no transgenic F2 off-spring was obtained from line #21. Thus, our studies on UREA-LacZ transgenic mice were mostly performed on line #22 and #26. For UREB-LacZ transgenic mice, three lines of germ-line transmitted founder mice #28, #81 and #96 were obtained for analysis. We first performed X-gal staining of transgenic mice at different embryonic stages to analyze the expression pattern of *LacZ* reporter gene. For either UREA-LacZ or UREB-LacZ transgenic embryos, despite that the expression levels of *LacZ* were varied among different founder lines, tissue-specific *LacZ* expression was observed. The results of *LacZ* expression patterns in developing UREA-LacZ and UREB-LacZ embryos were summarized in Table S1 and Table S2. These results implicated that, the *Nolz-1* related UREA and UREB *cis*-elements could serve as tissue-specific enhancers for controlling down-stream *LacZ* gene expression during embryogenesis.

### Preferential Expression of *LacZ* Reporter Gene in the Central Nervous System of UREA-LacZ Transgenic Embryos

#### Early onset of *LacZ* reporter gene expression in developing spinal cord

**Figure 2:**
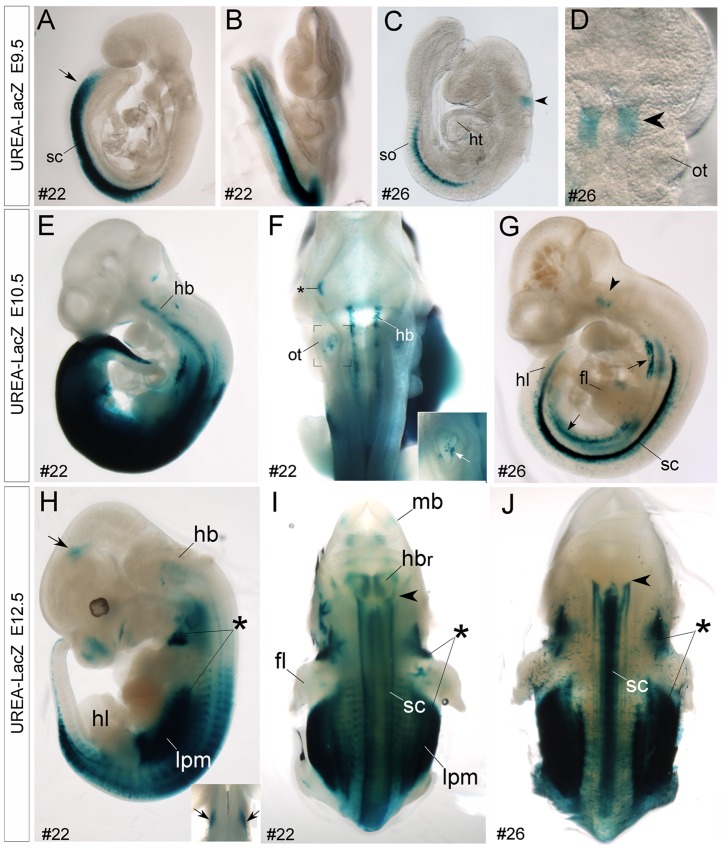
Detection of X-gal-positive signals in spinal cord and hindbrain of E9.5-E12.5 UERA–LacZ embryos. A–J: Whole mount X-gal staining of E9.5 (A–D), E10.5 (E–G) and E12.5 (H–J) UREA-LacZ embryos. Prominent X-gal-positive signals are detected in the spinal cord (sc) of either line #22 (A, B, E, F) or #26 (C, D, G) UREA-LacZ embryos at E9.5 and E10.5. Note that, this expression is earlier and stronger in line #22 than in line #26. Interestingly, in the hindbrain (hb) region, X-gal-positive signals are detected in a selective domain of hindbrain (arrowhead) in line #26 embryos at E9.5 (C, D) and E10.5 (G). Later at E12.5, the X-gal-positive signals expend into more caudal regions of hindbrain with a pattern of longitudinal columns (arrowhead in J indicates the anterior expression border). In line #22 hindbrain, longitudinal X-gal-positive signals are detected from E10.5 at more broad regions than line #26 (E, F). H, I: By E12.5, the X-gal-positive signals are also detected in the rostral part of hindbrain (hbr, H). Asterisks in H-J indicate strong X-gal-positive signals in the paraxial mesenchyme of embryos in both lines. Notably, X-gal-positive signals are also present in the lateral plate mesoderm (lpm, H, I; arrows in G) in both lines, and in otic vesicle (ot, F and white arrow in inset at high magnification) and midbrain region (mb, I; arrows in H and inset of H) of line #22 embryos. Asterisk in F indicates a non-specific signal. fl, forelimb bud; ht, heart; hl, hindlimb bud; so, somites.

The most impressive *LacZ* expression in UREA-LacZ transgenic embryos at early developmental stages was in the spinal cord and hindbrain regions. X*-*gal signals were observed as early as embryonic (E) day 9.5 in spinal cord *of UREA-LacZ transgenic embryos* (Figure 2A–C). Levels and regions of *LacZ* expression in spinal cord increased in both two founder lines during development. At E9.5, X-gal-positive signals were initially observed in the caudal half and in the middle region of spinal cord in line #22 (Figure 2A, B) and line #26 embryos (Figure 2C), respectively. Later after E10.5, the X-gal-positive signals were detected throughout the entire spinal cord in both two founder lines (Figure 2E, G–J and Figure S3B, D).

**Figure 3:**
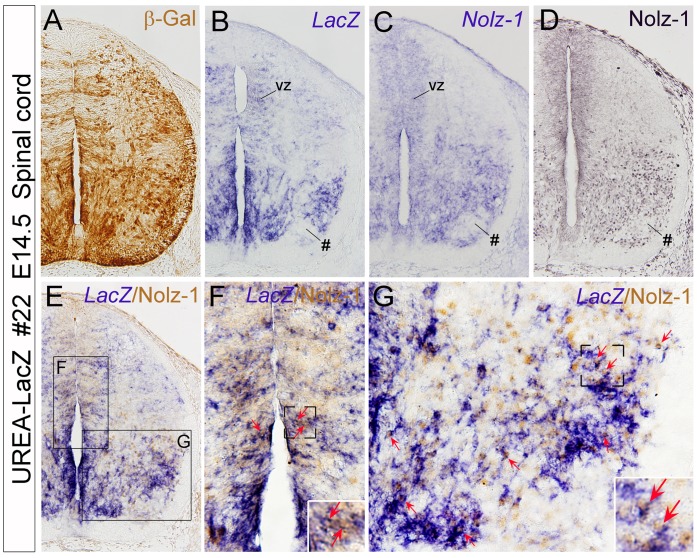
Detection of *LacZ* and *Nolz-1* co-expressing cells in spinal cord of UREA-LacZ embryos. A–D: Adjacent transverse sections of E14.5 spinal cord of line #22 UREA-LacZ embryos at the lumbar level. Expression patterns of β-gal protein (A) and *LacZ* mRNA (B) are similarly except that, in addition to soma, β-gal immunoreactivity is present in neurites, which results in broad expression pattern (A). Expression of *Nolz-1* at mRNA (C) and protein (D) levels are consistently detected in selective domains of spinal cord, including in the ventricular zone (vz) of dorsal and ventral spinal cord and medial and lateral regions of the ventral spinal cord where *LacZ* gene (B) is also expressed. A *LacZ* and *Nolz-1*-negative domain is present in the ventrolateral part of spinal cord (see # in B–D). E–G: Double labeling of *LacZ* mRNA and *Nolz-1* protein identifies many co-expressing cells (red arrows) in the ventricular zone (F) and in the medial and lateral domains of basal plate of spinal cord (G ). The boxed regions in E are shown at high magnification in F and G.

The expression levels of X-gal were lower in line #26 than in line #22 embryos, but the expression patterns in spinal cord at early developmental stages were similar in these two lines (Figure S4). In E13.5 spinal cord transverse sections, high levels of X-gal-positive signals were detected in the ventral regions adjacent to the midline (Figure S4). In addition, moderate levels of X-gal-positive signals were also detected in the lateral region of ventral and dorsal spinal cord of line#22 embryo (Figure S4A–D). Considering the possibility that the expression pattern of *LacZ* reporter gene might be different at mRNA and protein levels, we performed immunohistochemistry (IHC) of β-galactosidase (β-gal) protein, the *LacZ* gene encoded protein, and *LacZ* mRNA *in situ* hybridization (ISH) on adjacent sections of line #22 spinal cord at E14.5. The results showed that both *LacZ* mRNA and β-gal protein were detected in the ventricular zone of alar (dorsal) and basal (ventral) plates and also in some selective domains of differentiating neurons in ventral spinal cord (Figure 3A, B). Given the localization of β-gal protein in neurites, the β-gal-immunoreactive signals were distributed more broadly than the *LacZ* mRNA-positive signals (Figure 3A, B).

#### Detection of *LacZ* and *Nolz-1* co-expressing cells in UREA-LacZ embryonic spinal cord

We further compared the expression of *LacZ* mRNA with that of *Nolz-1* in E14.5 spinal cord. Interestingly, the expression pattern of *LacZ* mRNA was similar to that of endogenous *Nolz-1* gene both at mRNA and protein levels (Figure 3B–D). By double labeling analysis, we found many *LacZ* and *Nolz-1* co-expressing cells in ventricular zone (Figure 3F), in a domain adjacent to the midline and the lateral region of ventral spinal cord (Figure 3G) in line #22 UREA-LacZ embryo. The *LacZ*+/Nolz-1+ cells were observed at different levels of spinal cord along the anterior-posterior axis (Figure S5A–F). Furthermore, in line #26 UREA-LacZ embryo, within a domain adjacent to the midline of ventral spinal cord where *LacZ* was mainly expressed, many *LacZ* and *Nolz-1* co-expressing cells were also detected (Figure S5J, K).

Previous study has shown that *Nolz-1* is expressed in somatic motor neurons of embryonic spinal cord in chicken [16]. We performed double-labeling analysis of *LacZ* mRNA and Isl1/2, the pan-marker for spinal motor neurons, in E14.5 spinal cord of line #22 embryos. Only few LacZ+/Isl1/2+ cells were detected in the lateral region of ventral spinal cord (Figure S5G–I).

Taken together, our results suggest that the UREA *cis*-element may take part in directing endogenous *Nolz-1* expression in developing spinal cord.

#### Spatiotemporal dynamic expression of *LacZ* reporter gene in the developing hindbrain

In addition to the developing spinal cord, the *LacZ* reporter gene was prominently expressed in the developing hindbrain of UREA-LacZ embryos (Figure 2). The mouse *Nolz-1* and its zebrafish homologues, nlz1 and nlz2, are expressed in selective rhombomeres (i.e., r3, r5 and caudal hindbrain) and selective cell types in developing hindbrain [9], [14]. It was of particular interest that, in line #26 embryos in which X-gal expression was lower than line#22, X-gal-positive signals were observed with a rhombomere-specific pattern at E9.5 hindbrain. An X-gal-positive band located rostral to the otic vesicle was detected (Figure 2C, D). This expression pattern remained at E10.5 and E11.5 (Figure 2G and Figure S3B, C). Notably, in E11.5 hindbrain of line #21 F1 embryos which was the lost founder line, the X-gal-positive signals were observed in discontinuous segments of hindbrain (Figure S3A). Comparing the *LacZ* mRNA expression pattern with that of r4-specific marker, Hox2b, we found that the LacZ-expressing domain was located in r3 and r4 regions in E11.5 hindbrain of line #26 UREA-LacZ embryos (data not shown). Taken together, our results suggested that the UREA cis-element of *Nolz-1* might be also involved in control of rhombomere-selective expression of *Nolz-1* at early stages of hindbrain segmentation.

**Figure 4:**
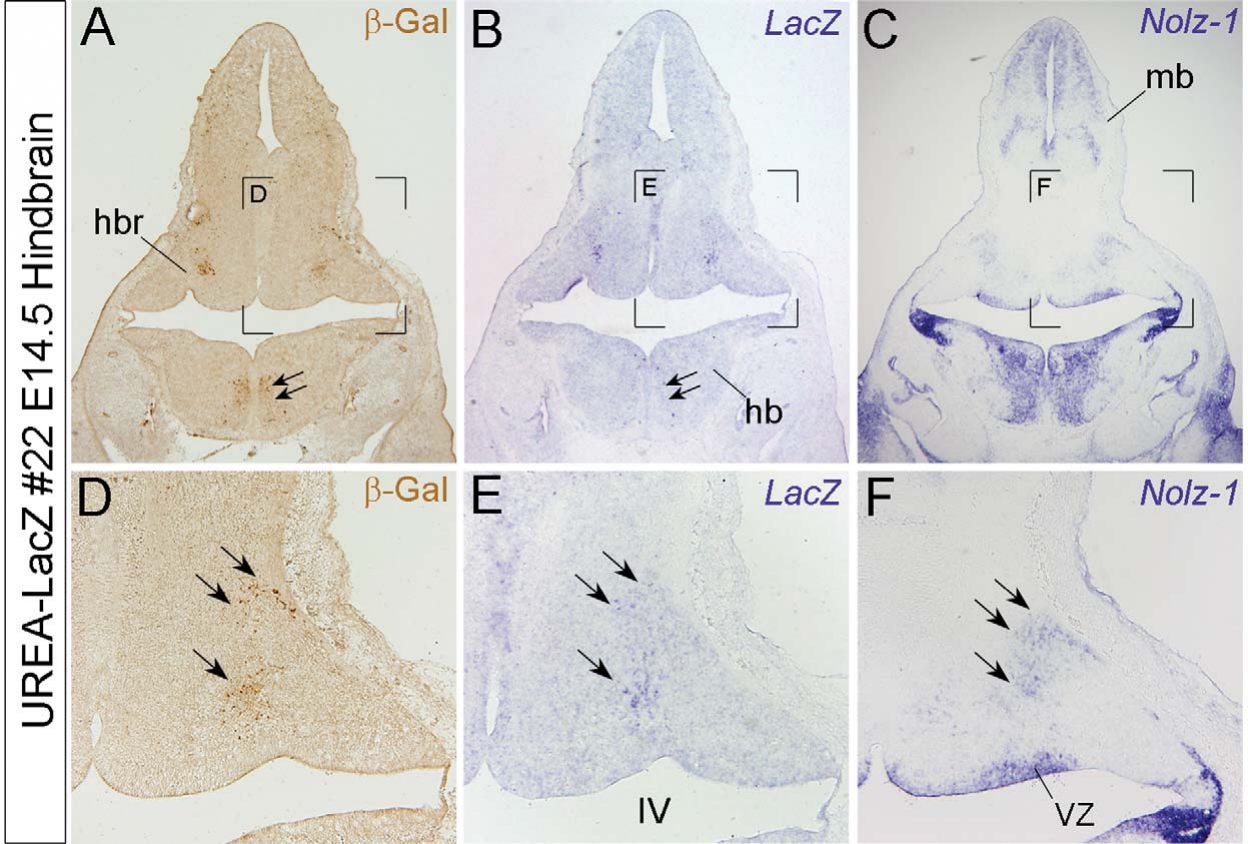
Detection of *LacZ* expression in a *Nolz-1* expressing domain in rostral hindbrain of line #22 UREA-LacZ embryos. A–C: Expressions of β-gal protein (A), *LacZ* mRNA (B), and *Nolz-1* mRNA (C) on coronal hindbrain sections of line#22 E14.5 UREA-LacZ embryo. D–E: High magnification views of the bracketed regions in A–C, respectively. Expressions of *LacZ* mRNA and β-gal protein are both detected in a differentiated domain of rostral hindbrain (triple arrows in D, E) where endogenous *Nolz-1* mRNA is expressed (F). *LacZ* expression is, however, absent in the nearby Nolz-1-expressing ventricular zone (vz, F). Double arrows in A and B indicate a domain in which β-gal expression is detected with little *LacZ* mRNA expression. IV, fourth ventricle; mb, midbrain.

At later stages, X-gal-positive signals were no longer restricted to specific rhombomeres. Instead, a specific pattern of longitudinal columns was found in the caudal hindbrain of line #26 embryos after E12.5 (Figure 2J and Figure S3E). Similar pattern of longitudinal columns was also observed in line#22, but at earlier stage of E10.5 (Figure 2E, F). In addition, X-gal-positive signals were also observed in the rostral part of hindbrain (hbr) of line #22 embryos after E12.5 (Figure 2I). Comparing *LacZ* at mRNA and protein levels in E14.5 line #22 hindbrain, we found that the *LacZ*-expressing domain in rostral hindbrain was correlated with the endogenous *Nolz-1* expressing domain (Figure 4). Note that the expressions of *LacZ* at mRNA and protein levels in the caudal hindbrain were, however, not consistent (double arrows, Figure 4 A, B).

**Figure 5:**
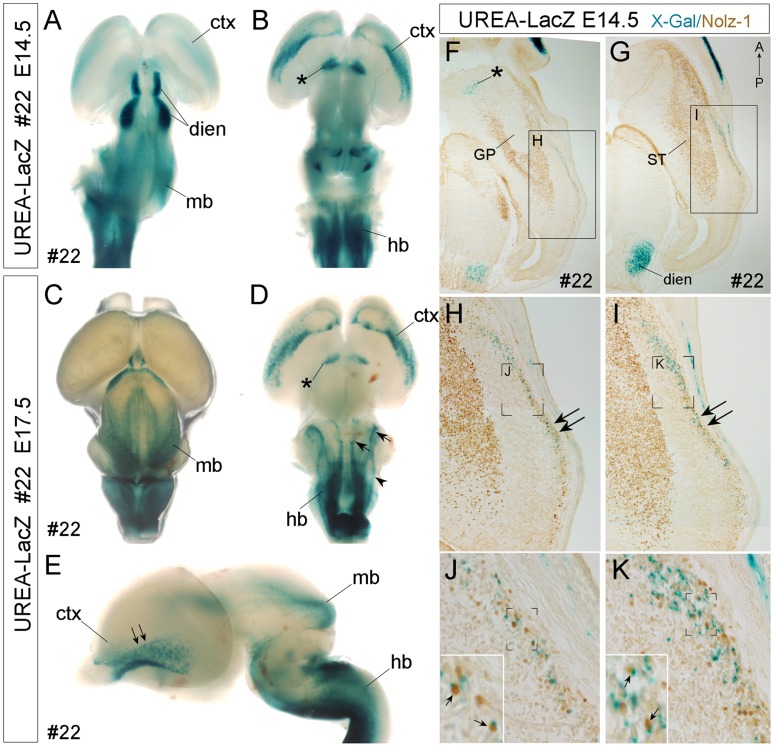
Detection of X-gal-positive signals in specific brain regions and *LacZ+*/Nolz-1+ cells in the ventral cortex of line #22 UERA-LacZ embryos. A–E: Whole mount X-gal staining of E14.5 (A, B) and E17.5 (C–E) embryonic brains. X-gal-positive signals are detected in the cerebral cortex (ctx, B, D), ventral forebrain (asterisk, B, D), diencephalon (dien, A), midbrain (mb, A, C, E) and hindbrain (hb, B, D, E) regions. D: Three X-gal-positive longitudinal columns (arrowhead, arrows) are found in the hindbrain. The arrows indicate the X-gal-positive columns which are also observed in the hindbrain of line #26 (Fig. S6), whereas the arrowhead indicates an additional X-gal-positive column found only in line #22. E: The double arrows indicate the ventral-to-dorsal gradient of X-gal-positive signals in the ventral cortex. A, C: dorsal view; B, D: ventral view; E: lateral view. F–K: Double labeling of X-gal-positive signal and *Nolz-1* protein on E14.5 horizontal forebrain sections of line#22 UREA-LacZ embryos. *Nolz-1* is expressed in the striatum (ST), but not in the globus pallidus (GP), nor in the X-gal-positive domains in ventral forebrain (asterisk, F) and diencephalon (dien, G). In the lateral cortex, a stream of X-gal-positive cells is observed. Within this cell stream, many X-gal-positive cortical cells co-expressing *Nolz-1* (small arrows in insets of J, K) are detected. Anterior is to the top of figures F–K. Figure F is at ventral level and Figure G is at more dorsal level. The boxed regions in F, G and the bracketed regions in H, I, J, K are shown at high magnification in H, I, J, K, and insets in J, K, respectively. IV, fourth ventricle; mb, midbrain.

Later at E17.5, two distinct and prominent X-gal-stained longitudinal columns were observed in the hindbrain of both lines (arrows, Figure 5D and Figure S6B). An extra lateral X-gal-positive strip was detected in the caudal part of E17.5 hindbrain in line #22 (arrowhead, Figure 5D). Although the expression patterns of *LacZ* at mRNA and protein levels were not consistent in developing caudal hindbrain, the pattern of *LacZ*-positive longitudinal columns was similar to that of endogenous *Nolz-1* [14].

Taken together, the rhomobere-specific expression at early segmental stages, the longitudinal columns expression pattern and the *Nolz-1* related rostral hindbrain expression of *LacZ* reporter gene did suggest an important role of UREA *cis*-element in directing *Nolz-1* expression in developing hindbrain.

#### Detection of *LacZ* and *Nolz-1* co-expressing cells in the ventrolateral cortex of developing telencephalon

The X-gal staining in line #22 UREA-LacZ embryos indicated that *LacZ* was also expressed in other brain regions, including the midbrain (Figure. 2H, J and Figure 5A, C, E) after E12.5, diencephalon (Figure 5A), ventral telencephalon (Figure 5B, D) and ventral cortex **(** Figure 5A, B, D, E) after E13.5. Double labeling of X-gal and *Nolz-1* protein in UREA-LacZ E14.5 brain showed that no *Nolz-1* expressing cells were observed in the X-gal-positive domains in the ventral forebrain (asterisk, Figure 5F), nor in diencephalon (dien, Figure 5G). Unexpectedly, many X-gal-positive cells co-expressing *Nolz-1* protein were found in the marginal mantle region of ventral cortex of line# 22 UREA-LacZ brain (Figure 5H–K; arrows in insets of Figure 5J, K).

The cortical X-gal-positive cells were found as early as E13.5∼E14.5 in the ventral cortex (Figure 5A, B). The number of X-gal-positive cortical cells increased, and these cells became dispersed dorsally as development progressed (Figure 5E and data not shown). Most X-gal-positive cortical cells co-expressing *Nolz-1* were observed at the middle and caudal levels (double arrows, Figure 5H, I) with few double positive cells present at the rostral level.

**Figure 6:**
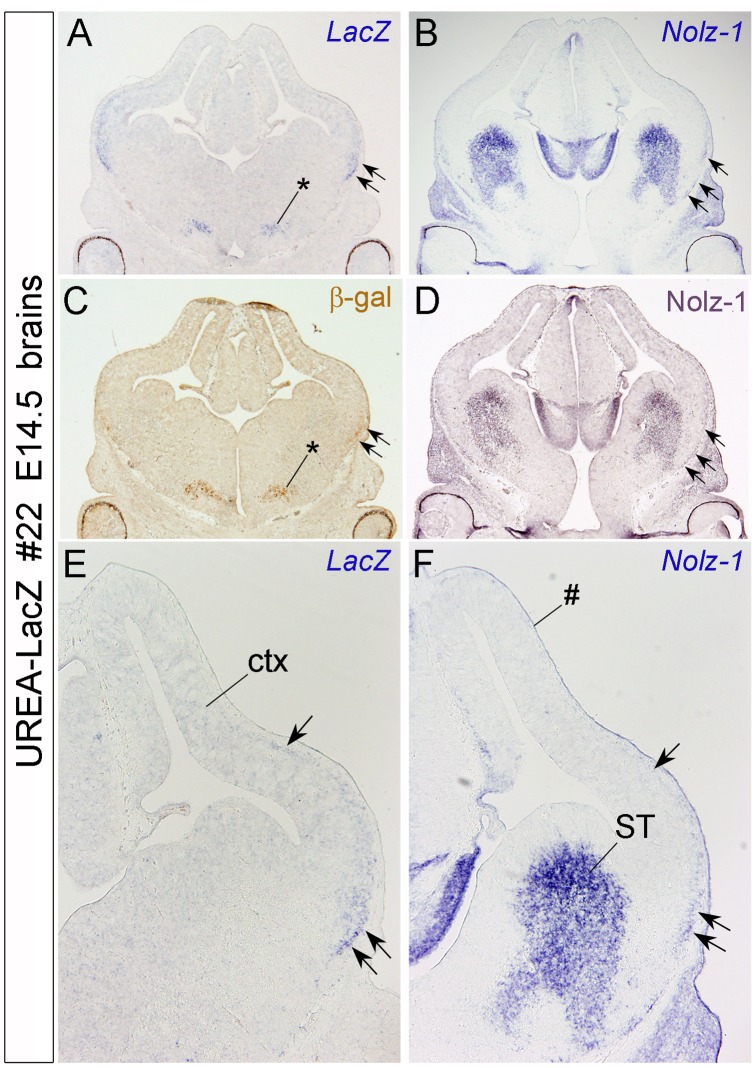
Detection of *LacZ* and *Nolz-1* mRNA expression in the ventrolateral cortex of E14.5 line #22 UREA-LacZ brain. A–D: Expression of *LacZ* mRNA (A), *Nolz-1* mRNA (B), β-gal (C) and *Nolz-1* protein (D) in coronal forebrain sections. Expressions of *LacZ* mRNA and β-gal protein are detected in a domain of ventral forebrain (asterisk, C) and in a strip of lateral cortex (ctx, double arrows in A, C and arrows in E). *Nolz-1* mRNA is also expressed in a similar lateral strip in cortex (arrows in B, D, F). Note that, more *LacZ*- and *Nolz-1*-expressing cells are present in the ventral part (double arrows, E, F) than in the dorsal part (single arrow, F) of the strip. ST, striatum. # indicates non-specific staining of meninges.

We previously reported that *Nolz-1* mRNA expression is expressed in diencephalon and midbrain, but not in the cerebral cortex of rat brain [13]. We therefore further compared the expression of *LacZ* and *Noz-1* at mRNA and protein levels in E14.5 cortex of line#22 UREA-LacZ embryo (Figure 6). *LacZ* mRNA and protein were detected in a domain of ventral forebrain (asterisk) and in the ventrolateral cortex (double arrows) as previously observed by X-gal staining (Figure 6A, C). *LacZ* mRNA was expressed as a lateral strip with a ventral-to-dorsal gradient in the ventrolateral cortex (Figure 6A, E). Interestingly, on adjacent sections, a similar lateral strip expression of *Nolz-1* either at mRNA or protein levels was also observed (arrows in Figure 6B, D, F). These findings not only revealed a new potential role of *Nolz-1* in cortical development, but they also suggest a role of UREA *cis*-element in regulating *Nolz-1* expression in this specific brain region.

In summary, the findings of UREA-LacZ embryos suggested that the UREA *cis*-element of *Nolz-1* gene was capable of directing *Nolz-1* expression in selective regions of developing central nervous system, including the spinal cord, hindbrain and specific cortical cells in the forebrain during development.

#### Detection of *LacZ* and *Nolz-1* co-expressing cells in non-neural tissues of UREA-LacZ embryos

In addition to the developing central nervous system, *LacZ* was also expressed in the lateral plate mesoderm (lpm, Figure 2G–J), otic vesicles (ot, Figure 2F and inset) and paraxial mesenchyme (rostral end at anterior region of forelimb buds and caudal end at the hindlimb buds) (asterisks, Figure 2H–J) in which endogenous *Nolz-1* was also expressed [14], [15]. Double labeling analysis detected many *LacZ* and *Nolz-1* co-expressing cells in the paraxial mesenchyme of UREA-LacZ embryos (Figure S5L and data not shown). These results suggested an important role of UREA in spatiotemporal regulation of *Nolz-1* expression in these tissue organs during development.

### Preferential Expression of *LacZ* Reporter gene in Selective Tissue/organs of UREB-LacZ Transgenic Embryos

**Figure 7:**
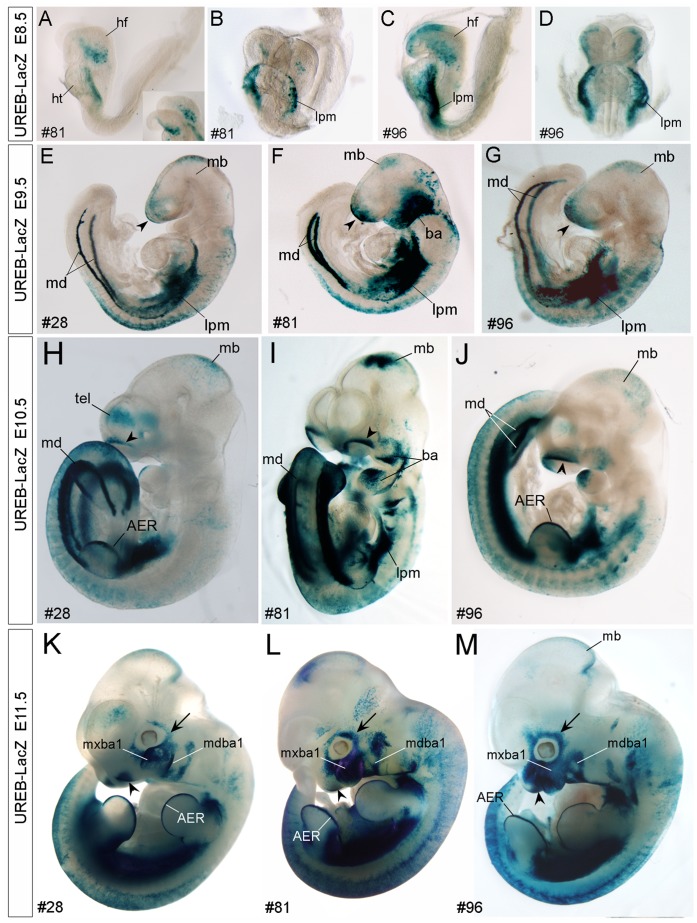
Detection of X-gal-positive signals in specific tissue organs of early developing UERB-LacZ embryos. A–D: X-gal signals are detected in the lateral plate mesoderm (lpm) and head fold structure (hf) of line #81 (A, B) and #96 (C, D) UREB-LacZ embryos at E8.5. E–G: At E9.5, the common regions containing X-gal-positive signals in all three lines of #28 (E), #81 (F) and #96 (G) are the anterior ridge of head (arrowhead), midbrain (mb), lateral plate mesoderm (lpm), mesonephric duct (md) and epidermis of embryonic bodies. Strong X-gal-positive signals are also detected in the branchial arches (ba) of line #81. H–M: Later at E10.5 (H–J) and E11.5 (K–M), prominent X-gal-positive signals are detected in the apical ectodermal ridge (AER, H, K, L) of limb buds and in olfactory pit (arrowhead, H–M). Although the expression levels are varied, selectively expression of *LacZ* in the branchial arches (ba) and its derivatives, including the mandibular (mdba1) and maxillary (mxba1) components of the first branchial arch, are consistently found in all three lines, (H–M), so as the selective expression of *LacZ* in the surrounding placodal tissues (arrow in H–M). A, C, E–H, J–M: side view; B, D, I: front view.

The *LacZ* expression in line #81 and #96 UREB-LacZ embryos was stronger and earlier than that in line #28 embryos. The overall expression patterns of *LacZ* were similar among these three lines (Figure 7). X-gal-positive signals were observed in more tissue organs in line#28 embryos than in line #81 and #96 embryos. X*-*gal-positive signals were initially observed in the lateral plate mesoderm (lpm) and head fold structure (hf) of line #81 and line #96 embryos (Figure 7A–D) at E8.5. X-gal-positive signals were persistently detected in lpm through early stages of embryogenesis (Figure 7A–J). X-gal-positive signals were not detected in E8.5 embryos of line #28 (data not shown).

At E9.5, X-gal-positive signals were observed in the anterior ridge of embryonic head, midbrain and mesonephric ducts (md) in all the three lines analyzed (Figure 7E–G). Later at E10.5 and E11.5, in addition to the lpm, md and midbrain, *X*-*gal-positive signals were also* observed in the apical ectodermal ridge (AER) of forelimb and hindlimb buds (Figure 7H–M).

**Figure 8:**
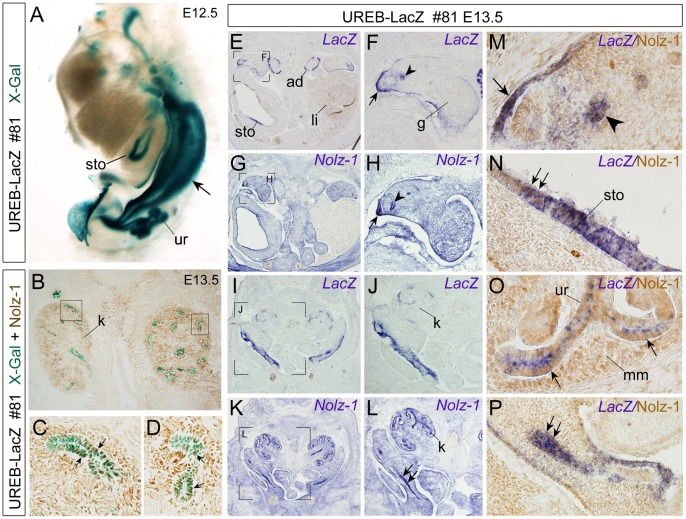
Detection of X-gal-positive signals in Nolz-1-expressing urogenital organs of UREB-LacZ embryos. A: By whole-mount staining, X-gal-positive signals are detected in the urogenital duct (arrow), ureteric tubules (ur) and stomach (sto) of E12.5 line #81 embryos. B–C: Many X-gal+/Nolz-1+ cells (arrows in C, D) were detected in some renal tubules of E13.5 kidney (k). The boxed regions in B are shown at high magnification in C, D. **E**–L: Expressions of *LacZ* (E, F, I, J) and *Nolz-1* mRNAs (G, H, K, L) in transverse sections of urogenital organs of E13.5 line #81 embryos. The bracketed regions in E, G, I, K are shown at high magnification in F, H, J, L, respectively. M–P: Double labeling of *LacZ* mRNA (purple) and *Nolz-1* protein (brown). *LacZ* and *Nolz-1* mRNAs are both detected in the genital ridge (arrow) and genital duct (arrowhead) (see also arrow and arrowhead in F, H). Many LacZ+/Nolz-1+ cells are detected in these genital tissues (M). *LacZ* mRNA is also detected in the adrenal gland (ad, E) and stomach (sto, E) of UREB-LacZ embryo. *LacZ* and *Nolz-1* co-expressing cells in stomach are detected (arrows, N). In the embryonic kidney (k), *LacZ* mRNA is mainly expressed in tubular cells (I, J, O), whereas *Nolz-1* mRNA (K, L) and protein are detected broadly both in the metanephric mesenchyme (mm, O) and in ureteric tubules (ur, O). *LacZ* and *Nolz-1* co-expressing cells in ureteric tub are detected (arrows, O). Double arrows in L and P indicate *Nolz-1* expression in urogenital tube where *LacZ* and *Nolz-1* co-expressing cells are found. g, gonads; li, liver; ad, adrenal gland.

Among the three lines of UREB-LacZ embryos, *LacZ* expression was consistently detected in the following tissues at mid-gestational stages: branchial arches and theirs derived tissues, the mandibular (mdba1) and maxillary (mxba1) components of the first branchial arch (Figure 7F, H–M and Figure S7A–C), epithelium of olfactory pit (arrowhead, Figure 7H–M and olf, Figure S7A–C), surrounding placodal tissue (single arrow, Figure 7K–M), epidermis of trunk (Figure 7E–M), stomach (Figure 8A), epithelium of lung and handplate (data not shown; Table S2). Moreover, X-gal-positive signals were detected in the cerebral cortex of E10.5–E11.5 telencephalon in line #28 embryos (Figure 7H, K and Figure S7A) and in the epiglottis and papilla of tongue in line #81 embryos after E12.5 (data not shown, Table S2).

Taken together, our results implicated that the UREB *cis*-element of *Nozl-1* functioned as a multiple tissue-specific enhancer in regulating *Nolz-1* expression during embryogenesis.

#### Detection of *LacZ* expression in *Nolz-1*-expressing urogenital system

Given that strong *LacZ* expression was detected in the mesonephric duct of UREB-LacZ embryos as early as E9.5 and was persistent through the mid-gestation stages (Figure 7E–M), we further analyzed the X-gal-positive signals in urogenital organs at later stages. The patterns of X-gal-positive signals among the three lines were similar in the branching ureteric tubules of kidney and in the genital ducts (Wollffian duct and Müllerian duct in male and female mice, respectively) of E14.5 UREB-LacZ embryos (Figure S7D–I). The X-gal-positive signals were also detected in the adrenal gland in line #28 and #81 (Figure S7D, E, G, H). Double labeling of *Nolz-1* protein and X-gal showed that *Nolz-1* was co-expressed in some X-gal-positive ureteric tubules of developing kidney (Figure 8B–D).

To our knowledge, *Nolz-1* expression has not yet been documented in the developing urogenital system. We were then interested to characterize endogenous *Nolz-1*, and compared its expression pattern to *LacZ* expression in the developing urogenital tissues of UREB-LacZ embryos. *LacZ* mRNA was detected in the adrenal gland, genital ridge, genital duct and some tubules in embryonic kidney as observed by X-gal staining in E13.5 embryos of line #81 (Figure 8E, F, I, J). *Nolz-1* mRNA expression was also detected in the genital ridge and genital duct (Figure 8G, H). Double labeling showed that many *LacZ*+/Nolz-1+ co-expressing cells in the genital ridge and genital tubes (Figure 8M, P). In the developing kidney, *Nolz-1* mRNA was detected in both metanephric mesenchyme and ureteric tubules (Figure 8K, L). Many *LacZ*+/Nolz-1+ co-expressing cells were detected in some renal tubules (Figure 8O). Co-expression of *LacZ* mRNA and *Nolz-1* protein in the epithelium of stomach was also observed (Figure 8N).

In summary, the analyses of UREB-LacZ embryos implicated that the UREB *cis*-element may play an important role in regulating *Nolz-1* expression in developing urogenital tissues, particularly in the tubular structures.

## Discussion

Previous genome-wide studies on non-coding sequences of the genomics found significant amounts of conserved non-coding sequences (CNEs) across the vertebrate lineage. Most of these CNEs are associated with genes implicated in transcriptional regulation and early development [19], [20], [21]. *In vivo* studies have shown that many of them containing enhancer activities direct reporter gene expression with tissue-specific patterns, which are highly correlated with the endogenous expression patterns of their associated genes [22], [23], [24]. These sequences are therefore characterized as the evolutionarily conserved *cis*-regulatory elements that play important roles in transcriptional regulation of their associated gene expression.

*Nolz-1,* as a CNEs-associated evolutionarily conserved gene, is dynamically expressed in specific tissue organs during embryogenesis [15], [20], [21], [22]. The functional conservation of mouse *Nolz-1* and its homologous genes has been implicated in the developing central nervous system, eyes and appendages. In the present study, we identified two evolutionarily conserved 5′ upstream *cis*-regulators of *Nolz-1,* which functioned as tissue-specific enhancers in regulating *Nolz-1* expression in selective tissue organs during embryogenesis.

### Similar *LacZ* Expression Patterns Among Different Reporter Mouse Lines Support Functional Roles of UREA and UREB Cis-elements as Tissue-specific Enhancers

Because the patterns of *LacZ* expression in many regions of developing tissue organs were correlated with that of endogenous *Nolz-1* expression, it is likely that the tissue-specific expressions of *LacZ* in transgenic embryos are regulated by *Nolz-1*-related UREA and UREB elements. The expressions of *LacZ* among different reporter mouse lines of UREA-LacZ and UREB-LacZ embryos were similar at most tissue organs, but there were also some differences. The differences between transgenic lines may be due to the position effects, *i.e*., the influences of *cis*-environments neighboring the transgenes.

For the UREA-LacZ mice, a pattern of broader and stronger expression of *LacZ* was observed in the central nervous system of line #22 than that in line #26. *LacZ* expression was detected in selective domains of telencephalon, diencephalon, midbrain, hindbrain and spinal cord in line #22 embryos, whereas in line #26 embryo, *LacZ* expression was only expressed in the hindbrain and spinal cord. It is notable that many *LacZ* expression regions in the spinal cord and hindbrain of line #22 were matched with the endogenous *Nolz-1* expressing domains, though in line #22, *LacZ* expression was detected in broader regions than that in line #26. These findings suggest a potential repression of reporter gene activity by surrounding *cis*-elements in line #26 UREA-LacZ mice. Alternatively, the enhancer activity of UREA may be weak in line #26. Conceivably, the cooperation of UREA with other *cis-*elements is required for precisely directing *Nolz-1* expression in spinal cord and hindbrain.

For the UREB-LacZ mice, the *LacZ* expression patterns were more consistent among the three reporter mouse lines though the expression levels were varied. Selective expression of *LacZ* in the olfactory pit, branchial arches and its derivatives, surrounding placodal tissues, midbrain, apical ectodermal ridge of limb buds, tubule structures of urogenital tissues, lateral plate mesoderm and epidermis of embryonic bodies were consistently found in the three mouse lines. Minor differences in the pattern of *LacZ* expression were found in some tissues, including the additional cortex expression in line #28 and the epiglottis and tongues expression in line #81. These results suggest that there is only little influence by *cis*-elements neighboring the transgenes on embryonic expression among different lines of UREB-LacZ embryos.

### Important Roles of UREA Cis-element for Regulating *Nolz-1* Expression in Developing Spinal Cord, Hindbrain and Ventrolateral Cortex

The UREA element effectively directed *LacZ* reporter gene expression in developing spinal cord and hindbrain. In the developing spinal cord, *Nolz-1* was expressed in several regions, including post-mitotic domains of ventral spinal cord and the ventricular zone of dorsal and ventral spinal cord [16]. Interestingly, the expression pattern of *LacZ* in spinal cord of line #22 embryos, the founder line with broad *LacZ* expression, was similar to that of *Nolz-1* expression at both mRNA and protein levels. These results implicate a crucial role of the UREA cis-element in regulating *Nolz-1* expression in developing spinal cord.

In the developing hindbrain, *LacZ* was expressed in discontinuous segmented hindbrain at early stages and also in specific domains and longitudinal columns at mid-gestational stages in both of the two mouse lines of UREA-LacZ embryos. This spatiotemporal dynamic expression pattern was similar to that of endogenous *Nolz-1* expression [14]. Our studies implicate a potential role of UREA in regulating *Nolz-1* expression in hindbrain development, particularly in the rostral hindbrain region where both *LacZ* mRNA and β-gal protein expressions are correlated with the endogenous *Nolz-1* expression. In the caudal hindbrain region, the expressions of *LacZ* mRNA and protein were not completely identical, which may be due to the fact that β-gal protein is relatively more stable than *LacZ* mRNA in the developing hindbrain.

In the developing forebrain at mid-gestational stages, we, unexpectedly, found *Nolz-1* expression in the marginal zone of ventral parts of cerebral cortex in which *LacZ* mRNA was also expressed in line #22 UREA-LacZ embryos. Many X-gal-positive cells co-expressing *Nolz-1* were found in UREA-LacZ ventral cortex, which suggested a role of UREA in regulating selective expression of *Nolz-1* in these ventral cortical cells. Because *LacZ* was expressed along a ventral-to-dorsal gradient in the ventral cortex, it raises the possibility that these *LacZ* and *Nolz-1* co-expressing cells are tangentially migrating cells. They might be rostral migratory stream (RMS) cells. However, *Nolz-1* is not expressed in Er81-positive dorsal lateral ganglionic eminence (dLGE) which gives rise to the RMS cells (unpublished observation) [25]. Alternatively, they might be tangentially migrating cortical interneurons from the medial and caudal ganglionic eminences (MGE and CGE), though *Nolz-1* is not expressed in the MGE except the corridor cells region (unpublished observation) [25], [26]. Several transcriptional factors highly expressed in the MGE and CGE are known to be required for generation of distinct subtypes of cortical interneurons [26]. Whether *Nolz-1* and *LacZ* co-expressing cells in the ventral cortex of UREA-LacZ brain are tangentially migrating cortical interneurons or they are other types of migrating cells remains to be clarified.

### Important Roles of UREB cis-element for Regulating *Nolz-1* Expression in Selective Developing Tissue Organs Including the Tubule Structures of Urogential Organs

Unlike the nervous tissue preferentially driven activity of UREA, UREB directed *LacZ* expression mainly in developing non-neural tissue organs, which included the branchial arches and its derivatives, *olfactory pit*, AER of the limb and the urogenital tissue organs, where endogenous *Nolz-1* was also expressed [15]. In the present study, we demonstrated that *Nolz-1* was expressed in developing urogenital tissues, including the genital ducts, *metanephric mesenchyme* and ureteric tubules. Double labeling of *LacZ* and *Nolz-1* detected many co-expressing cells in the genital ridge, mesonephric duct, ureteric tubule of E13.5 UREB-LacZ embryos. Taken together, our study suggests that UREB *cis*-element is likely to play a crucial role in controlling endogenous *Nolz-1* expression in these selective tissue organs. Notably, both UREA and UREB elements directed strong expression of *LacZ* in the lateral plate mesoderm where *Nolz-1* was also expressed [*15*].

The mammal *Nolz-1/Zfp503* and *Zfp703* are the orthologues of zebrafish *nlz-2* and *nlz-1* genes, respectively. These two paralogues genes are identified in almost all vertebrates whose genomes have been sequenced and annotated. In *amphioxus*, a species which holds a central place in the evolutionary diversity when considering the origin of vertebrates, these two genes correspond to a single locus, *amphioxus ZNF503/ZNF703*. Interestingly, besides being conserved in the orthologue *nlz-2* locus, a small region of UREB was also conserved in the *nlz-1* locus. This suggests that the UREB *cis*-element may be important for regulating *Nolz-1* homologous genes expression, and thus have been conserved following genome duplication in the vertebrate lineage. Indeed, the expression pattern of *LacZ* in the UREB-LacZ embryos partially reflected the endogenous *Nolz-1* expression pattern, particularly in the branchial arches (pharyngeal tissues), early developing limb buds and the urogenital system.

Previous study has demonstrated tissue-specific enhancer activity of a 489 bp-composite element, which is composed by five short *amphioxus* conserved non-coding *cis*-elememts (aCNEs) of human *ZNF503* [27]. Our analysis of sequences alignments showed that the sequences of 13^th^ to 96^th^ (84 bp) and 204^th^ to 285^th^ (82 bp) bp of UREB shared high identities with the sequences of 325^th^ to 243^th^ (95%) and 243^th^ to 162^th^ (99%) bp, respectively, of this combinatory aCNEs in reversed orientation. Consistently, *LacZ* reporter gene expression in UREB-LacZ and human ZNF503 CNE *LacZ* reporter mice was observed in developing midbrain, AER of the developing limb buds and the pharyngeal tissues [25]. It suggests that the key *cis*-elements for regulating the reporter gene expression in these three tissues were located in these two short sequence motifs of UREB.

### Spatiotemporal Expression of *Nolz-1* in Selective Tissue Organs During Embryogenesis may be Regulated by Multiple Evolutionarily Conserved cis-elements

The mismatched patterns of *LacZ* expression and endogenous *Nolz-1* expression in some tissue organs of UREA-LcaZ and UREB-LacZ transgenic embryos indicate that other regulatory sequences are necessary to fully recapitulate endogenous *Nolz-1* expression. Some other *cis*-elements with repression activities may be required for proper *Nolz-1* expression, because *LacZ* expression is present in some tissues in which *Nolz-1* expression was not detected, such as some domains in the ventral telencephalon and diencephalon of line #22 UREA-LacZ embryos and the adrenal gland of UREB-LacZ embryos. Conversely, no *LacZ* expression was observed in some regions of developing brain where endogenous *Nolz-1* was expressed, particularly in the developing striatum in which *Nolz-1* is highly expressed [13]. These results implicate that some other *cis*-elements are required to coordinate with UREA and/or UREB to precisely regulate endogenous *Nolz-1* expression during embryogenesis.

In addition to the two short evolutionarily conserved *cis-*elements of Pz1 and Pz2 in the potential promoter region, we also found a conserved *cis*-element in 3′ UTR of *Nolz-1*. These uncharacterized *cis*-elements may play a role in regulation of endogenous *Nolz-1* expression. It is of interest that we have previously indentified a functional retinoic response element (RARE) located at the 5′ end of 1.1 kb P1 promoter region [13]. Given the important role of retinoic acid in regulating striatal development, the *cis*-regulatory elements controlling striatal *Nolz-1* expression may reside in the putative promoter region.

In summary, our present study has identified two evolutionarily conserved 5′ upstream *cis*-elements that are capable of directing *Nolz-1* expression in specific neural and non-neural regions during embryonic development. Further studies will be required to unveil the conserved transcriptional mechanisms underlying spatiotemporal expression of *Nolz-1* in different regions and tissues during development.

## Supporting Information

Figure S1 **Nucleotide sequences and sequence alignments of** ***Nolz-1*** **UREA element.**
(DOC)

Figure S2 **Nucleotide sequences and sequence alignments of** ***Nolz-1*** **UREB element.**
(DOC)

Figure S3 **Whole mount X-gal staining of E11.5 UREA-LacZ embryos.** A: In embryos of line #21, X-gal-positive signals are selectively detected in two discontinuous domains of hindbrain (arrowheads) and in the spinal cord (sc). B–C: In embryos of line#26, X-gal-positive signals are detected in a selective domain of hindbrain (arrowhead), in spinal cord (sc) and in the lateral plate mesoderm (arrows). D–G: In addition to the spinal cord (sc) and caudal hindbrain (hb), X-gal-positive signals are also detected in the otic vesicle (ot, E, G) and a specific domain at oral region (arrows, F) in line #22 embryos. A, B, D: lateral view; C, E: back view; F: front view; G: transverse view of truncated hindbrain (hb) of UREA-LacZ embryo.
(TIF)

Figure S4 **Detection of X-gal-positive signals in spinal cord of E13.5 UREA-LacZ embryos.** A–K, transverse sections of E13.5 spinal cord. Panels are arranged from the rostral (A, E, I) to caudal levels (D, H, K). A, B at the cervical level; B–C, F–G and I–J at the thoracic level; D, H, K at the lumbar level. X-gal-positive signals are detected in ventral spinal cord of #22 (lightly stained, A–D) and #26 (lightly stained, E–H; darkly stained, I–K) at all levels along the rostral-to-caudal axis in embryonic spinal cord. X-gal-positive signals in line #26 are mainly detected in a domain near the floor plate (E–K), whereas in line #22, X-gal-positive signals are found in broader regions, including the lateral domains of ventral spinal cord (A–D). Note that at caudal levels, X-gal-positive signals are also observed in scattered cells in dorsal spinal cord with moderate expression levels (D).
(TIF)

Figure S5 **Double labeling of** ***LacZ*** **mRNA and** ***Nolz-1*** **protein or Isl1/2 protein in E14.5 spinal cord of UREA-LacZ embryos.** A–C, D–F: Double labeling of *LacZ* mRNA (purple) and *Nolz-1* protein (brown) in spinal cord of line #22 UREA-LacZ embryos. D, E, F are high magnification views of A, B, C, respectively, in the ventrolateral spinal cord. The # symbols indicate the regions where many *LacZ* and *Nolz-1* co-expressing spinal neurons are observed (D–F). J–L: Double labeling of *LacZ* mRNA (purple) and Isl1/2 (brown) proteins. A few cells expressing *LacZ* mRNA at low level co-express Isl1/2 (single arrow in inset of H). J–K: Double labeling of *LacZ* mRNA (purple) and *Nolz-1* protein (brown) in spinal cord of line #26 UREA-LacZ embryos. Many LacZ-positive cells co-expressing *Nolz-1* (arrows in inset of K) are observed. D, J are transverse sections of spinal cord at the level of heart; B, E, H at the level of upper abdominal; C, F, I, J–L at the level of lower abdominal.
(TIF)

Figure S6 **Whole mount X-gal staining of E15.5 brain of line #26 UREA-LacZ embryo.** Two X-gal-positive longitudinal columns (arrows, B), similar to that observed in line #22 (Fig. 4D) are detected in line #26 hindbrain (hb). A: dorsal view; B: ventral view; C: side view.
(TIF)

Figure S7 **Whole mount X-gal staining of E11.5 frontal heads and E14.5 urogenital organs of UREB-LacZ embryos.** A–C: Front views of the whole mount stained UREB-LacZ embryonic heads at E11.5 show that X-gal-positive signals are detected in the olfactory pit (olf) and the mandibular (mdba1) and maxillary (mxba1) components of the first branchial arch in lines #28 (A), #81 (B) and #96 (C) embryos. In line #28 embryo, X-gal-positive signals are also detected in the cortex (ctx, A). D–I: The patterns of X-gal-positive signals are similar in the urogenital organs among line #28 (D, G), #81 (E, H) and #96 (F, I) of E14.5 UREB-LacZ embryos. Strong X-gal signals are detected in the Wolffian duct (Wd, D) and Müllerian duct (Md, H) of female (D–F) and male (G–I) genital tubules. X-gal-positive signals are also detected in the adrenal gland (a, F) and ureteric tubules (ut, I) in kidney (k, G, I), but not in the gonads (testis, t, F) of E14.5 UREB-LacZ embryos.
(TIF)

Table S1 ***LacZ*** **expression in developing UREA-LacZ mouse embryos.**
(DOC)

Table S2 ***LacZ*** **expression in developing UREB-LacZ mouse embryos.**
(DOC)

## References

[1] Nakamura M, Runko AP, Sagerstrom CG (2004) A novel subfamily of zinc finger genes involved in embryonic development. J Cell Biochem 93: 887–895. 15449319 10.1002/jcb.20255

[2] Holland DG, Burleigh A, Git A, Goldgraben MA, Perez-Mancera PA, et al (2011) ZNF703 is a common Luminal B breast cancer oncogene that differentially regulates luminal and basal progenitors in human mammary epithelium. EMBO Mol Med 3: 167–180. 21337521 10.1002/emmm.201100122 PMC3395113

[3] Slorach EM, Chou J, Werb Z (2011) Zeppo1 is a novel metastasis promoter represses E-cadherin expression and regulated p120-catenin isoform expression and location. Genes Dev 25: 471–484. 21317240 10.1101/gad.1998111 PMC3049288

[4] Zhao X, Yang Y, Fitch DH, Herman MA (2002) TLP-1 is an asymmetric cell fate determinant that responds to Wnt signals and controls male tail tip morphogenesis in *C. elegans* . Development 129: 1497–1508. 11880358 10.1242/dev.129.6.1497

[5] Weihe U, Dorfman R, Wernet MF, Cohen SM, Milan M (2004) Proximodistal subdivision of *Drosophila* legs and wings: the elbow-no ocelli gene complex. Development 131: 767–774. 14757638 10.1242/dev.00979

[6] Cheah PY, Meng YB, Yang X, Kimbrell D, Ashburner M, et al (1994) The Drosophila l(2)35Ba/nocA gene encodes a putative Zn finger protein involved in the development of the embryonic brain and the adult ocellar structures. Mol Cell Biol 14: 1487–1499. 8289824 10.1128/mcb.14.2.1487-1499.1994 PMC358504

[7] Dorfman R, Glazer L, Weihe U, Wernet MF, Shilo BZ (2002) Elbow and Noc define a family of zinc finger proteins controlling morphogenesis of specific tracheal branches. Development 129: 3585–3596. 12117809 10.1242/dev.129.15.3585

[8] Tseng ASK, Hariharan IK (2002) An overexpression screen in *Drosophila* for genes that restricted growth or cell-cycle progression in the developing eye. Genetics 162: 229–243. 12242236 10.1093/genetics/162.1.229 PMC1462252

[9] Hoyle J, Tang YP, Wiellette EL, Wardle FC, Sive H (2004) nlz gene family is required for hindbrain patterning in the zebrafish. Dev Dyn 229: 835–846. 15042707 10.1002/dvdy.20001

[10] Runko AP, Sagerstrom CG (2003) Nlz belongs to a family of zinc-finger-containing repressors and controls segmental gene expression in the zebrafish hindbrain. Dev Biol 262: 254–267. 14550789 10.1016/s0012-1606(03)00388-9

[11] Runko AP, Sagerstrom CG (2004) Isolation of nlz2 and characterization of essential domains in Nlz family proteins. J Biol Chem 279: 11917–11925. 14709556 10.1074/jbc.M310076200

[12] Brown JD, Duttac S, Bharti K, Bonner RF, Munson PJ, et al (2009) Expression profiling during ocular development identifies 2 Nlz genes with a critical role in optic fissure closure. Proc Natl Acad Sci U S A 106: 1462–1467. 19171890 10.1073/pnas.0812017106 PMC2631080

[13] Chang CW, Tsai CW, Wang HF, Tsai HC, Chen HY, et al (2004) Identification of a developmentally regulated striatum-enriched zinc-finger gene, Nolz-1, in the mammalian brain. Proc Natl Acad Sci U S A 101: 2613–2618. 14983057 10.1073/pnas.0308645100 PMC356998

[14] Chang SLY, Yan YT, Shi YL, Liu YC, Takahashi H, et al (2011) Region- and cell type-selective expression of the evolutionarily conserved Nolz-1/zfp503 gene in the developing mouse hindbrain. Gene Exp Pattern 11: 525–532. 10.1016/j.gep.2011.09.003 21945624

[15] McGlinn E, Richman JM, Metzis V, Town L, Butterfield NC, et al (2008) Expression of the NET family member Zfp503 is regulated by hedgehog and BMP signaling in the limb. Dev Dyn 237: 1172–1182. 18351672 10.1002/dvdy.21508

[16] Ji SJ, Periz G, Sockanathan S (2009) Nolz1 is induced by retinoid signals and controls motoneuron subtype identity through distinct repressor activities. Development 136: 231–240. 19056829 10.1242/dev.028043 PMC2685968

[17] Zerucha T, Stühmer T, Hatch G, Park BK, Long Q, et al (2000) A highly conserved enhancer in the *Dlx5/Dlx6* intergenic region is the site of cross-regulatory Interactions between *Dlx* genes in the embryonic forebrain. J Neurosci 20: 709–721. 10632600 10.1523/JNEUROSCI.20-02-00709.2000 PMC6772408

[18] Yannaki E, Tubb J, Aker M, Stamatoyannopoulos G, Emery DW (2002) Topological constraints governing the use of the chicken HS4 chromatin Insulator in oncoretrovirus vectors. Mol Ther 5: 589–598. 11991750 10.1006/mthe.2002.0582

[19] Sandelin A, Bailey P, Bruce S, Engstrom PG, Klos JM, et al (2004) Array of ultraconserved non-coding regions span the loci of key developmental genes in vertebrate genome. BMC Genomics 5: 99. 15613238 10.1186/1471-2164-5-99 PMC544600

[20] Woolfe A, Goodson M, Goode DK, Snell P, McEwen GK, et al (2005) Highly conserved non-coding sequences are associated with vertebrate development. PLoS Biol 3: e7. 15630479 10.1371/journal.pbio.0030007 PMC526512

[21] Woolfe A, Elgar G (2007) Comparative genomics using Fugu reveals insights into regulatory subfunctionalization. Genome Biol 8: R53. 17428329 10.1186/gb-2007-8-4-r53 PMC1896008

[22] McEwen GK, Woolfe A, Goode D, Vavouri T, Callaway H, et al (2006) Ancient duplicated conserved noncoding elements in vertebrates: a genomic and functional analysis. Genome Res 16: 451–465. 16533910 10.1101/gr.4143406 PMC1457030

[23] Abbasi AA, Paparidis Z, Malik S, Bangs F, Schmidt A (2010) et?al (2010) Human intronic enhancers control distinct sub-domains of *Gli3* expression during mouse CNS and limb development. BMC Dev Biol 10: 44. 20426846 10.1186/1471-213X-10-44 PMC2875213

[24] Lee AP, Brenner S, Venkatesh B (2011) Mouse transgenesis identifies conserved functional enhancers and cis-regulatory motif in the vertebrate LIM homeobox Gene Lhx2 locus. PLoS ONE. 6(5): e20088 doi:10.1371/journal.pone.0020088. 10.1371/journal.pone.0020088 PMC3100342 21629789

[25] Stenman J, Toresson H, Campbell K (2003) Identification of two distinct progenitor populations in the lateral ganglionic eminence: implications for striatal and olfactory bulb neurogenesis. J Neurosci 23: 167–174. 12514213 10.1523/JNEUROSCI.23-01-00167.2003 PMC6742158

[26] Zaki PA, Quinn JC, Price DJ (2003) Mouse models of telencephalic development. Curr Opin Genet Dev 13: 423–437. 12888017 10.1016/s0959-437x(03)00084-4

[27] Holland LZ, Albalat R, Azumi K, Benito-Gutierrez E, Blow MJ, et al (2008) The amphioxus genome illuminates vertebrate origins and cephalochordate biology. Genome Res 18: 1100–1111. 18562680 10.1101/gr.073676.107 PMC2493399

