# Supplementary material for: Identification of Two Evolutionarily Conserved 5' cis-Elements Involved in Regulating Spatiotemporal Expression of Nolz-1 during Mouse Embryogenesis
Source: PLoS One. 2013 Jan 22;8(1):e54485. doi: 10.1371/journal.pone.0054485 (PMC3551757; doi:10.1371/journal.pone.0054485)
Supplement: Figure S1 — Nucleotide sequences and sequence alignments of Nolz-1 UREA element. (DOC) [file pone.0054485.s001.doc]

**Figure S1. Nucleotide sequences and sequence alignments of *Nolz-1* UREA element**

**A. Nucleotide sequence of mouse *Nolz-1* UREA element**

CCCTCCATTT ACCGGTCTCC AGTTTACCAC TCCCACGCCC CAGGGGCCCC 50

GCGCCTCCTC CCCTCCCCCC ATCCCACCCC ACTCCCGCTC TCCCGGTCGC 100

TTTAAATAAT GATATTTGCA TGCAAGGAAC GATTCATAAA TATGTCAGGG 150

CCGGTGAAAT ATAGGCAACA TTTCAAACTT TCTATTTAAA AAACATGAAT 200

TATGGCTGCG AAAAATGTGT TCCCATTTAA AGGCGATACG AAGTATTTGG 250

TGTCTCGTCC CCGGGCCCAT CCATCATGCA GACAATAAAG AGAGTTTTTC 300

GCCCTGACAC TCGGGATTTA TGGGCGCCCT TCTCTGCCCT CATCACTCAC 350

CCACTGAC TCGGGATTTA TGGGCGCCCT TCTCTGCCCT CATCACTCAC 355

The nucleotides in blue color indicate the zebrafish *nlz2* conserved sequences in mouse *Nolz-1* UREA element.

**B. DNA sequence alignments between mouse *Nolz-1* UREA element and the conserved genomic sequence of zebrafish *nlz2***

Identities = Identities = 97/111 (87%), Gaps = 5/111 (5%)

UREA 185 ATTTAAAAAACATGAATTATGGCTGCGAAAAATGTGTTCCCATTTAAAGGCGATACGAAG 244

||||||| || |||||||||| | ||||| || |||||||||| | || | ||

Nlz2 ATTTAAATAATATGAATTATGACCCACTAAAATATGCTCCCATTTAAGGATAATGCCGAG

UREA 245 TATTTGGTGTCTCGTCCCCGGGCCCATCCATCATGCAGACAATAAAGAGAGTTTTTCGCC 304

| ||| || ||| | | ||| ||| | |||||||||||||||||||| | ||| |

Nlz2 TGTTTTGTATCTGATTCAGAGGCTCATTCGTCATGCAGACAATAAAGAGAATATTTTTAC

UREA 305 CTGACACTCGGGATTTATGGGCGCCCTTCTCTGCCCTCATCACTCACCCA 354

| || || | |||||||| ||||||||||| || || |||||| ||

Nlz2 CCGATGCTGGAAATTTATGGTTACCCTTCTCTGCTCTAATAACTCACGCA
