# Supplementary material for: Identification of Two Evolutionarily Conserved 5' cis-Elements Involved in Regulating Spatiotemporal Expression of Nolz-1 during Mouse Embryogenesis
Source: PLoS One. 2013 Jan 22;8(1):e54485. doi: 10.1371/journal.pone.0054485 (PMC3551757; doi:10.1371/journal.pone.0054485)
Supplement: Figure S2 — Nucleotide sequences and sequence alignments of Nolz-1 UREB element. (DOC) [file pone.0054485.s002.doc]

**Figure S2. Nucleotide sequences and sequence alignments of *Nolz-1* UREB element**

**A. Nucleotide sequence of mouse *Nolz-1* UREB element**

CAGTCCAGGA CTCTTAGGGC GCTCTGGGCC AGGCTTTCCA ACTTGCCGGG 50

CCTGACCTGT CAGGCGGATT ATCTTCGAGG GAGATTAATA GGGGAGGCGG 100

GCTGCAATAA TAATCGTTTT GTTTGATGTG ACAACTCTGA TAGGCGTTGA 150

TTTACTTACA AACTGATAGG CTTTTAATTG AGCGCCTCCG CCGAGCCAGA 200

GATGAAAGGG AAGCGGTGGT GAAAGGCGGC CCGCCTGCCC TCCGGCCTCA 250

ATACTGATTA GCCATCTCGA CAGTGAATAG TTCAAGGCAT TTTCAAACTT 300

TTTTCCTCGG CTCTCTTCCC TTCCGCCTTC TTCCCTCCAC CTCCTCCTCA 350

AATATCTGCC ATGCCCACAC CCTTTTCTTC TTCCCTCCAC CTCCTCCTCA 376

The blue letters indicated the conserved sequences of *Nolz-1* UREB element and its zebrafish *nlz2* orthologue. The underlined sequences indicate the sequences which are also conserved in *nlz1* locus.

**B. DNA sequence alignments between mouse *Nolz-1* UREB element and the conserved genomic sequence of zebrafish *nlz2***

Identities = 226/261 (87%), Gaps = 16/261 (6%)

UREB 53 TGACCTGTCAGGCGGATTATCTTC-GAGGGAGATTAATAGG-GGAGGCGGGCTGCAATAA 111

|||||||||| |||||||| | | || ||||||||| || ||||||||| ||||||||

Nlz2 TGACCTGTCAAGCGGATTA-CCGCAGAAAGAGATTAAT-GGTGGAGGCGGGTTGCAATAA

UREB 112 TAATCGTTTTGTTTGATGTGACAACTCTGATAGGCGTTGATTTACTTACAAACTGATAGG 171

|||||||||||||||||||||||||| |||||||||||||||||||||||||||||||||

Nlz2 TAATCGTTTTGTTTGATGTGACAACTTTGATAGGCGTTGATTTACTTACAAACTGATAGG

UREB 172 CTTTTAATTGAGCGCCTCCG-CCGAGCCAGAGATGAAAGGGAA-GCGG-TGGTGAAAGGC 228

||||||||||||||||||| || || |||||||||||| || ||| | ||||| ||

Nlz2 CTTTTAATTGAGCGCCTCCATCC-AGTGAGAGATGAAAGGCAAAGCGCAT--TGAAAAGC

UREB 229 GGCCCGCCTGCCCTCCG-GCCTCAATACTGATTAGCCATCTC-GACAGTGAATAGTTCAA 286

||||| ||||| | | ||||||||||||||||||| |||| | | |||||||||||||

Nlz2 TGCCCGATTGCCC-CGGAGCCTCAATACTGATTAGCCGTCTCAG-CGGTGAATAGTTCAA

UREB 287 GGCATTTTCAAACTT-TTTTC 305

|||||||| |||||| |||||

Nlz2 GGCATTTT-AAACTTCTTTTC

**C. DNA sequence alignments between mouse *Nolz-1* UREA element and the conserved genomic sequence of zebrafish *nlz1* gene.**

Identities = 97/111 (87%), Gaps = 5/111 (5%)

UREB 81 GAGATTAAT-AGGGGAGGCGG-GCTGCAATAATAATCGTTTTGTTTGATGTGACAACTCT 139

||||||||| ||| | ||||| || | ||||||| || ||||||||| ||||||| ||

Nlz1 GAGATTAATGAGGTG-GGCGGAGCAG-TATAATAAACG-TTTGTTTGAAGTGACAAGGCT

UREB 140 GATAGGCGTTGATTTACTTACAAACTGATAGGCTTTTAATTGAGCGCCTCC 189

|||||| ||||||||||||||| ||||||||||||||||||||||||||||

Nlz1 GATAGGTGTTGATTTACTTACACACTGATAGGCTTTTAATTGAGCGCCTCC
