# Supplementary figures and images for: Identification of Two Evolutionarily Conserved 5' cis-Elements Involved in Regulating Spatiotemporal Expression of Nolz-1 during Mouse Embryogenesis
Source: PLoS One. 2013 Jan 22;8(1):e54485. doi: 10.1371/journal.pone.0054485 (PMC3551757; doi:10.1371/journal.pone.0054485)

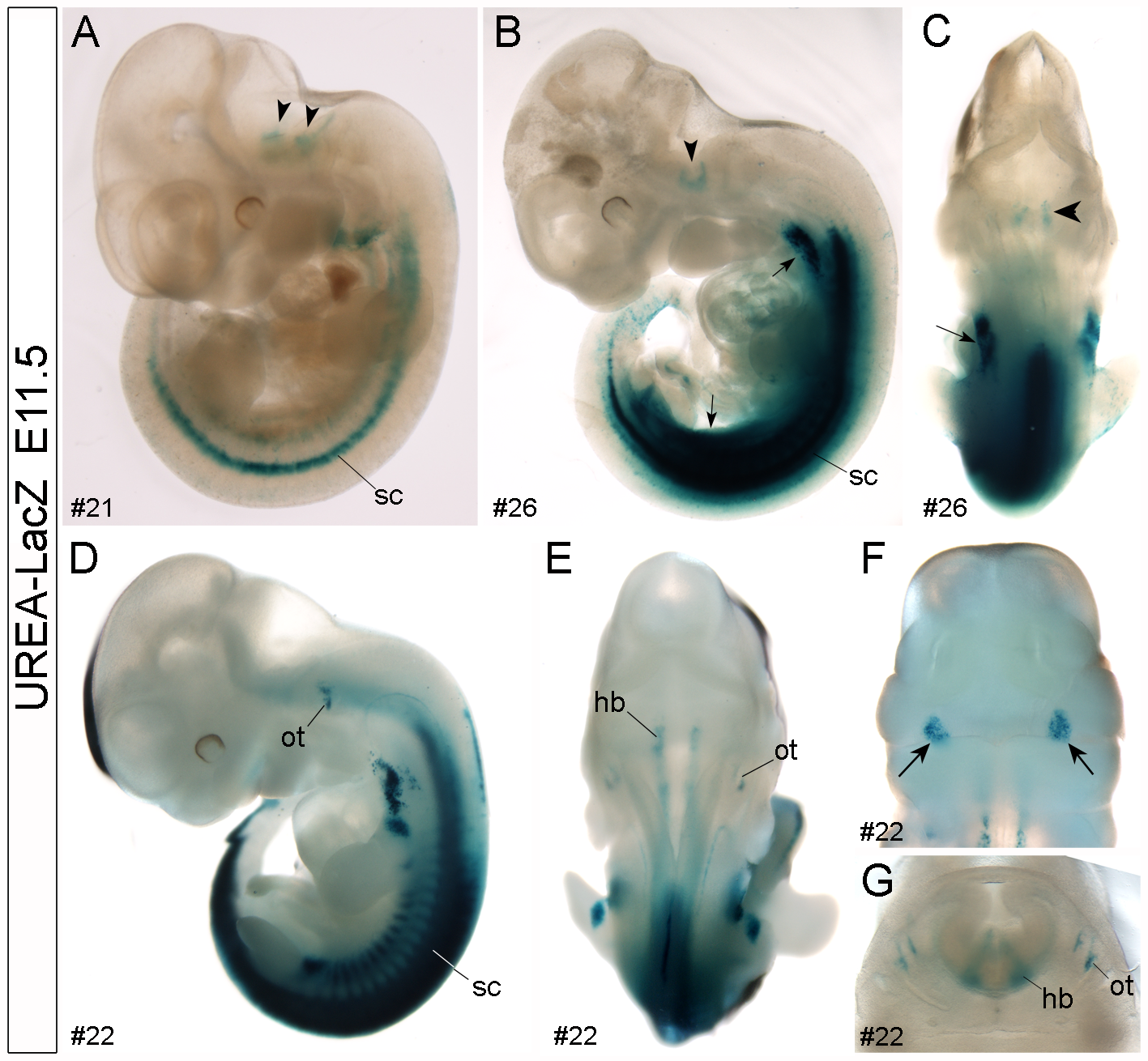

Supplement: Figure S3 — Whole mount X-gal staining of E11.5 UREA-LacZ embryos. A: In embryos of line #21, X-gal-positive signals are selectively detected in two discontinuous domains of hindbrain (arrowheads) and in the spinal cord (sc). B–C: In embryos of line#26, X-gal-positive signals are detected in a selective domain of hindbrain (arrowhead), in spinal cord (sc) and in the lateral plate mesoderm (arrows). D–G: In addition to the spinal cord (sc) and caudal hindbrain (hb), X-gal-positive signals are also detected in the otic vesicle (ot, E, G) and a specific domain at oral region (arrows, F) in line #22 embryos. A, B, D: lateral view; C, E: back view; F: front view; G: transverse view of truncated hindbrain (hb) of UREA-LacZ embryo. (TIF) [file pone.0054485.s003.tif]

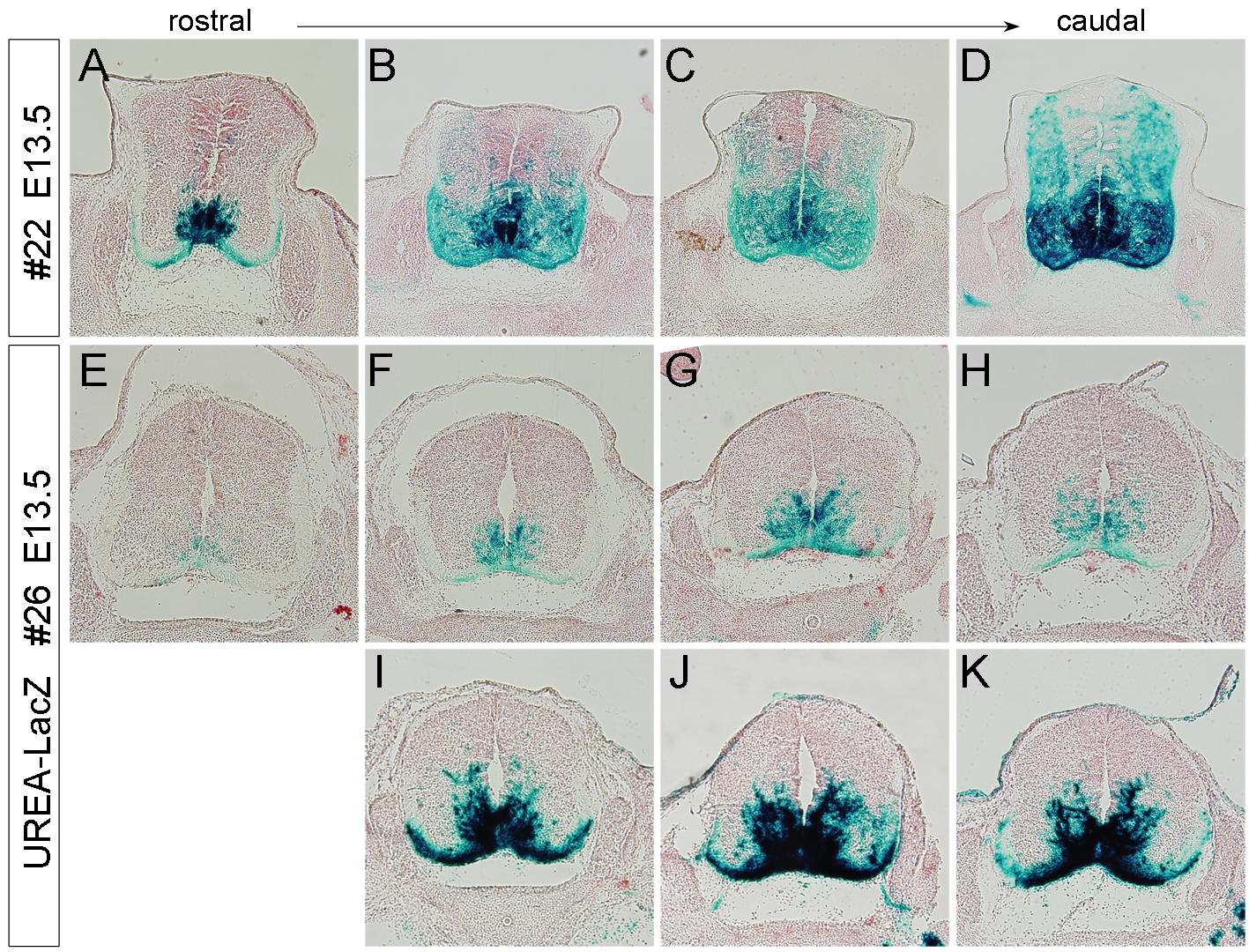

Supplement: Figure S4 — Detection of X-gal-positive signals in spinal cord of E13.5 UREA-LacZ embryos. A–K, transverse sections of E13.5 spinal cord. Panels are arranged from the rostral (A, E, I) to caudal levels (D, H, K). A, B at the cervical level; B–C, F–G and I–J at the thoracic level; D, H, K at the lumbar level. X-gal-positive signals are detected in ventral spinal cord of #22 (lightly stained, A–D) and #26 (lightly stained, E–H; darkly stained, I–K) at all levels along the rostral-to-caudal axis in embryonic spinal cord. X-gal-positive signals in line #26 are mainly detected in a domain near the floor plate (E–K), whereas in line #22, X-gal-positive signals are found in broader regions, including the lateral domains of ventral spinal cord (A–D). Note that at caudal levels, X-gal-positive signals are also observed in scattered cells in dorsal spinal cord with moderate expression levels (D). (TIF) [file pone.0054485.s004.tif]

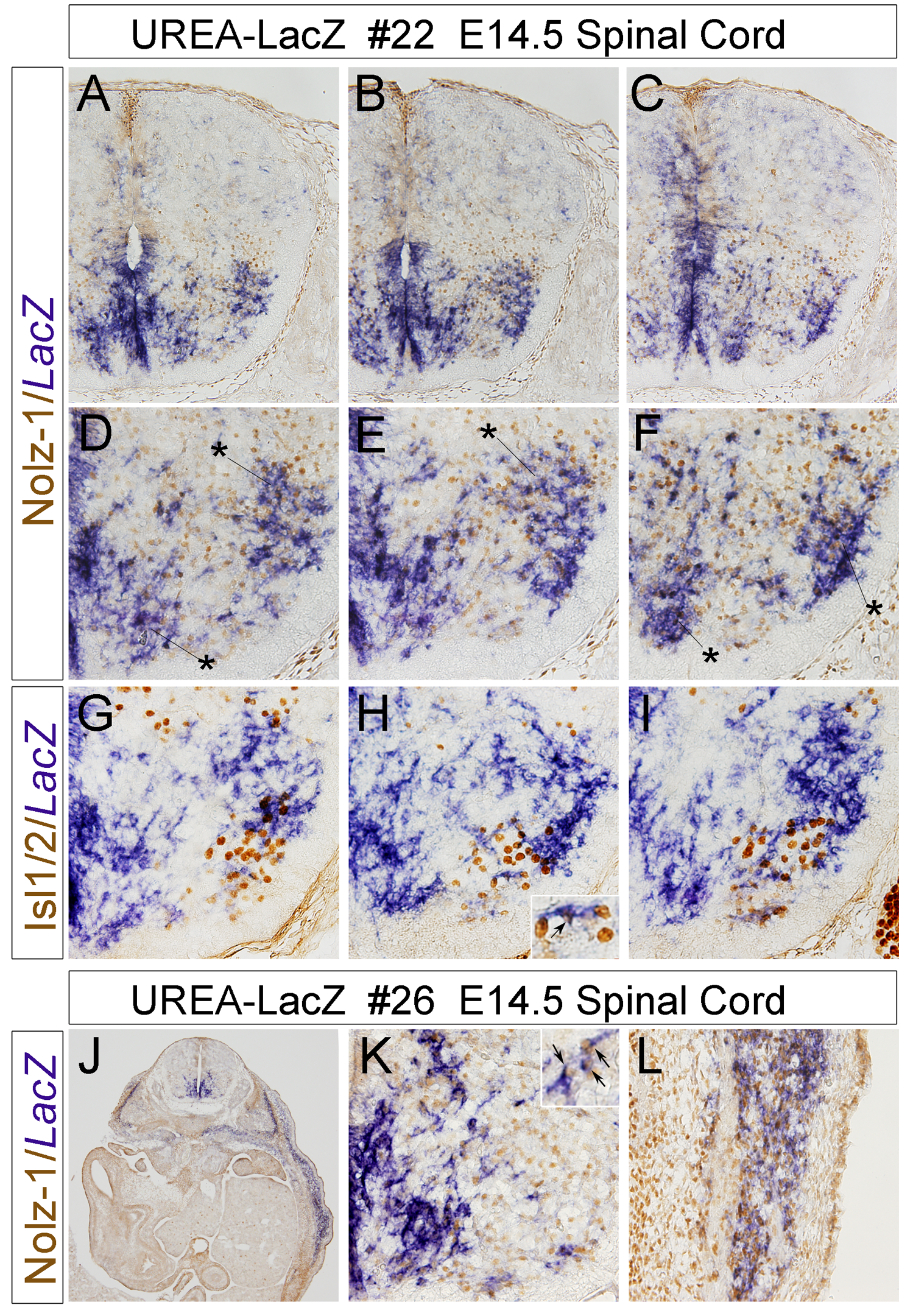

Supplement: Figure S5 — Double labeling of LacZ mRNA and Nolz-1 protein or Isl1/2 protein in E14.5 spinal cord of UREA-LacZ embryos. A–C, D–F: Double labeling of LacZ mRNA (purple) and Nolz-1 protein (brown) in spinal cord of line #22 UREA-LacZ embryos. D, E, F are high magnification views of A, B, C, respectively, in the ventrolateral spinal cord. The # symbols indicate the regions where many LacZ and Nolz-1 co-expressing spinal neurons are observed (D–F). J–L: Double labeling of LacZ mRNA (purple) and Isl1/2 (brown) proteins. A few cells expressing LacZ mRNA at low level co-express Isl1/2 (single arrow in inset of H). J–K: Double labeling of LacZ mRNA (purple) and Nolz-1 protein (brown) in spinal cord of line #26 UREA-LacZ embryos. Many LacZ-positive cells co-expressing Nolz-1 (arrows in inset of K) are observed. D, J are transverse sections of spinal cord at the level of heart; B, E, H at the level of upper abdominal; C, F, I, J–L at the level of lower abdominal. (TIF) [file pone.0054485.s005.tif]

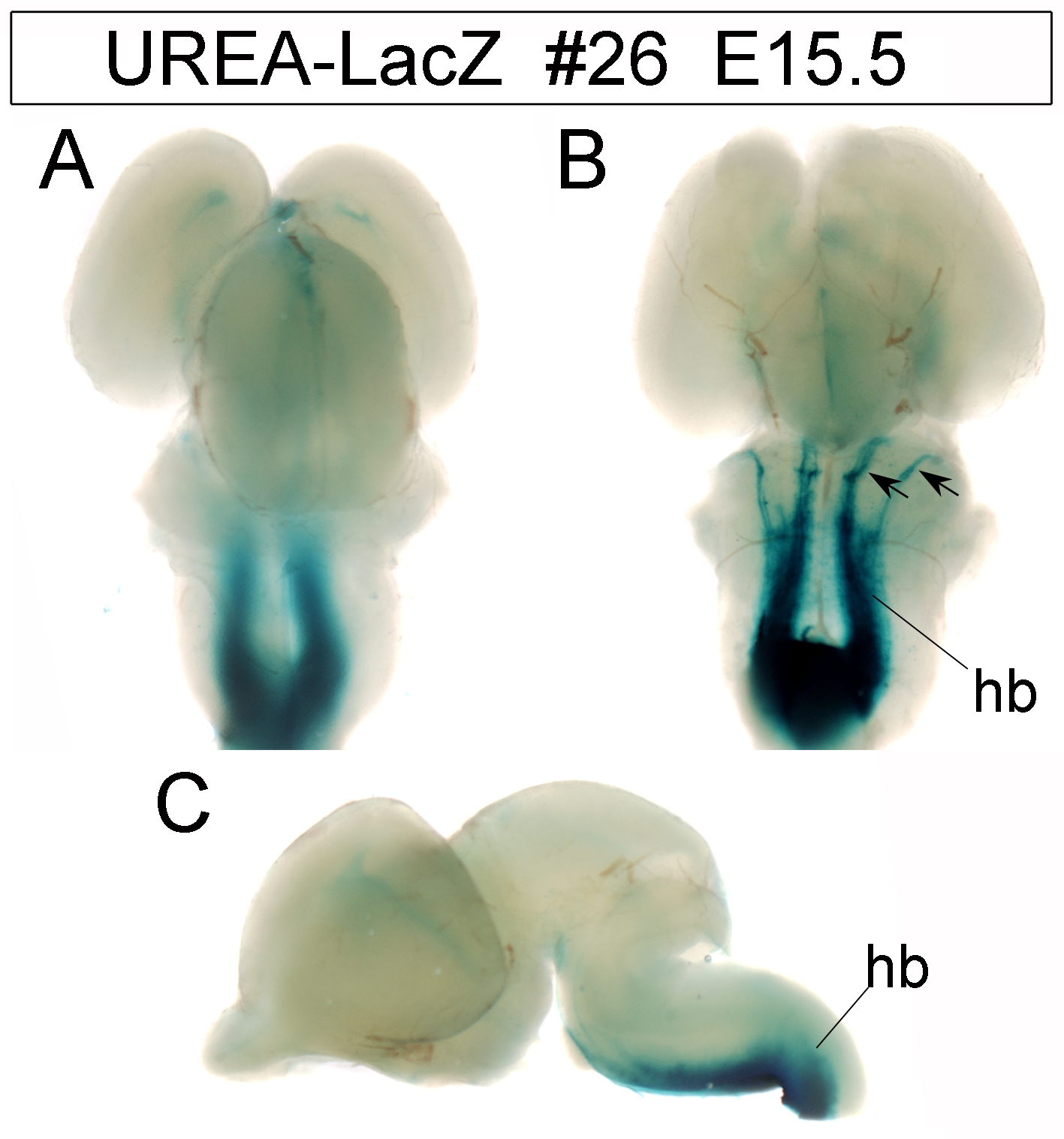

Supplement: Figure S6 — Whole mount X-gal staining of E15.5 brain of line #26 UREA-LacZ embryo. Two X-gal-positive longitudinal columns (arrows, B), similar to that observed in line #22 (Fig. 4D) are detected in line #26 hindbrain (hb). A: dorsal view; B: ventral view; C: side view. (TIF) [file pone.0054485.s006.tif]

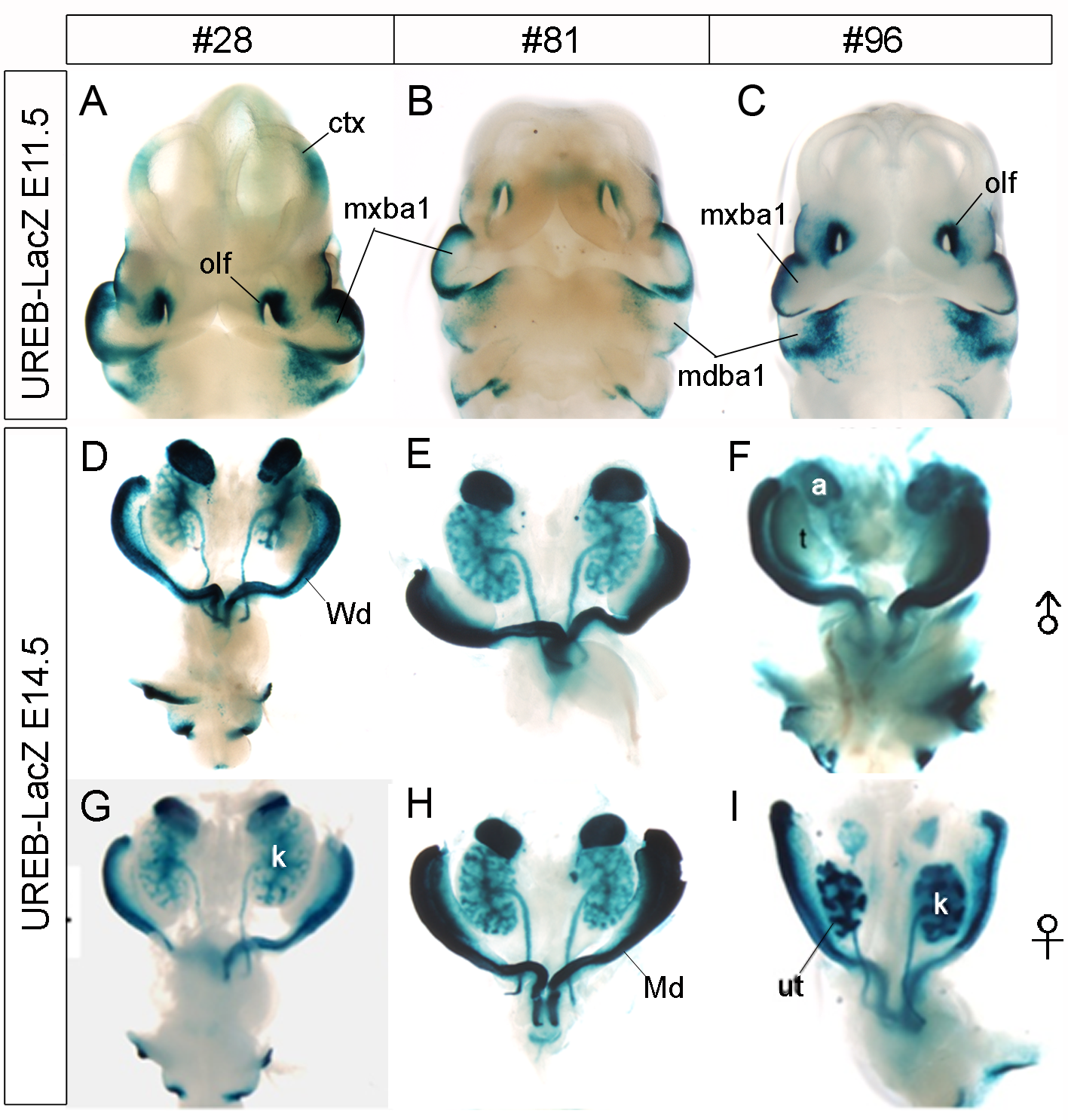

Supplement: Figure S7 — Whole mount X-gal staining of E11.5 frontal heads and E14.5 urogenital organs of UREB-LacZ embryos. A–C: Front views of the whole mount stained UREB-LacZ embryonic heads at E11.5 show that X-gal-positive signals are detected in the olfactory pit (olf) and the mandibular (mdba1) and maxillary (mxba1) components of the first branchial arch in lines #28 (A), #81 (B) and #96 (C) embryos. In line #28 embryo, X-gal-positive signals are also detected in the cortex (ctx, A). D–I: The patterns of X-gal-positive signals are similar in the urogenital organs among line #28 (D, G), #81 (E, H) and #96 (F, I) of E14.5 UREB-LacZ embryos. Strong X-gal signals are detected in the Wolffian duct (Wd, D) and Müllerian duct (Md, H) of female (D–F) and male (G–I) genital tubules. X-gal-positive signals are also detected in the adrenal gland (a, F) and ureteric tubules (ut, I) in kidney (k, G, I), but not in the gonads (testis, t, F) of E14.5 UREB-LacZ embryos. (TIF) [file pone.0054485.s007.tif]
