# Supplementary material for: Identification of Two Evolutionarily Conserved 5' cis-Elements Involved in Regulating Spatiotemporal Expression of Nolz-1 during Mouse Embryogenesis
Source: PLoS One. 2013 Jan 22;8(1):e54485. doi: 10.1371/journal.pone.0054485 (PMC3551757; doi:10.1371/journal.pone.0054485)
Supplement: Table S1 — LacZ expression in developing UREA-LacZ mouse embryos. (DOC) [file pone.0054485.s008.doc]

**Table S1. *LacZ* expression in developing UREA-LacZ mouse embryos**

| **Embryonic stage** | E9.5 | | E10.5 | | E11.5 | | E12.5 | | E13.5 | | E14.5 | | 15.5 | |
| --- | --- | --- | --- | --- | --- | --- | --- | --- | --- | --- | --- | --- | --- | --- |
| **UREA-LacZ founder line** | **22** | **26** | **22** | **26** | **22** | **26** | **22** | **26** | **22** | **26** | **22** | **26** | **22** | **26** |
| **Central Nervous System** | | | | | | | | | | | | | | |
| forebrain | - | - | - | - | - | - | + | - | + | - | + | - | + | - |
| midbrain | - | - | - | - | - | - | + | - | + | - | + | - | + | - |
| hindbrain | + | + | + | + | + | + | + | + | + | + | + | + | + | + |
| spinal cord | + | + | + | + | + | + | + | + | + | + | + | + | + | + |
| **Head** | | | | | | | | | | | | | | |
| otic vesicle / inner ear | - | - | + | - | + | - | + | - | + | - | + | - | + | - |
| nose (lateral-nasal process mesenchyme) | - | - | - | - | + | - | + | - | + | - | + | - | + | - |
| oral region (epithelium) | - | - | - | - | + | - | + | - | + | - | + | - | + | - |
| **Trunk** | | | | | | | | | | | | | | |
| lateral plate mesoderm | - | - | + | +/- | + | + | + | + | + | + | - | - | - | - |
| paraxial mesenchyme | + | - | + | +/- | + | + | + | + | + | + | + | + | + | + |
| **Epidermis** | | | | | | | | | | | | | | |
| head region | - | - | - | - | - | - | - | - | - | - | - | + | - | + |
| trunk region | - | - | - | - | - | +/- | - | + | - | + | - | + | - | + |
| limb region | - | - | - | + | - | + | - | + | - | + | - | + | - | + |
