# Supplementary material for: Identification of Two Evolutionarily Conserved 5' cis-Elements Involved in Regulating Spatiotemporal Expression of Nolz-1 during Mouse Embryogenesis
Source: PLoS One. 2013 Jan 22;8(1):e54485. doi: 10.1371/journal.pone.0054485 (PMC3551757; doi:10.1371/journal.pone.0054485)
Supplement: Table S2 — LacZ expression in developing UREB-LacZ mouse embryos. (DOC) [file pone.0054485.s009.doc]

**Table S2. *LacZ* expression in developing UREB-LacZ mouse embryos**

**Embryonic stage**E10.5E11.5E12.5E13.5E14.5E15.5**UREB-LacZ founder line**288196288196288196288196288196288196**Central Nervous System**telencephelon+-++-++-----------midbrain++++++++++++------**Head**surrounding placodal tissue---+++++++++++++++olfactory pit (epithelium) ++++++++++++++++++maxillary component ++++++++++++++++++mandibular component++++++++++++++++++epiglottis-------+--+--+--+-tongue (papilla ) -------+--+--+--+-**Trunk** epidermis of trunk++++++++++++++++++lung (epithelium)+++++++++---------stomach ++++++++++++++++++adrenal gland++++++++++++++++++kidney metanephros++++++++++++++++++ureteric tube++++++++++++++++++Wolffian duct++++++++++++++++++Müllerian duct++++++++++++++++++**Appendages**AER of limb buds+++++++++++++/-+/-+/----handplate / palm------++++++++++++
